# Trop-2 Is a Determinant of Breast Cancer Survival

**DOI:** 10.1371/journal.pone.0096993

**Published:** 2014-05-13

**Authors:** Federico Ambrogi, Marco Fornili, Patrizia Boracchi, Marco Trerotola, Valeria Relli, Pasquale Simeone, Rossana La Sorda, Rossano Lattanzio, Patrizia Querzoli, Massimo Pedriali, Mauro Piantelli, Elia Biganzoli, Saverio Alberti

**Affiliations:** 1 Unit of Medical Statistics, Biometry and Bioinformatics “Giulio A. Maccacaro”, Department of Clinical Sciences and Community Health, University of Milan, Milano, Italy; 2 Unit of Cancer Pathology, Department of Biomedical Sciences and CeSI, Fondazione ‘G. D'Annunzio’, University of Chieti, Chieti, Italy; 3 MediaPharma s.r.l., CeSI, University ‘G. D'Annunzio’, Chieti, Italy; 4 Section of Surgical Pathology, Department of Experimental and Diagnostic Medicine, University of Ferrara, Ferrara, Italy; 5 Fondazione IRCCS, Istituto Nazionale Tumori, Milano, Italy; 6 Department of Neurosciences, Imaging and Clinical Sciences – Physiology and Physiopathology, University of Chieti, Chieti, Italy; 7 Oncoxx Biotech s.r.l., Chieti, Italy; University of Dundee, United Kingdom

## Abstract

Trop-2 is a calcium signal transducer that drives tumor growth. Anti-Trop-2 antibodies with selective reactivity versus Trop-2 maturation stages allowed to identify two different pools of Trop-2, one localized in the cell membrane and one in the cytoplasm. Of note, membrane-localized/functional Trop-2 was found to be differentially associated with determinants of tumor aggressiveness and distinct breast cancer subgroups. These findings candidated Trop-2 states to having an impact on cancer progression. We tested this model in breast cancer. A large, consecutive human breast cancer case series (702 cases; 8 years median follow-up) was analyzed by immunohistochemistry with anti-Trop-2 antibodies with selective reactivity for cytoplasmic-retained versus functional, membrane-associated Trop-2. We show that membrane localization of Trop-2 is an unfavorable prognostic factor for overall survival (1+ *versus* 0 for all deaths: hazard ratio, 1.63; P = 0.04), whereas intracellular Trop-2 has a favorable impact on prognosis, with an adjusted hazard ratio for all deaths of 0.48 (high *versus* low; P = 0.003). A corresponding impact of intracellular Trop-2 was found on disease relapse (high versus low: hazard ratio, 0.51; P = 0.004). Altogether, we demonstrate that the Trop-2 activation states are critical determinants of tumor progression and are powerful indicators of breast cancer patients survival.

## Introduction

Breast cancer is the most frequent malignancy in women, with almost 800 new cases per year per 100,000 women. Breast tumors are markedly heterogeneous in their biological aggressiveness, response to therapy, and prognosis [1]–[4]. Even patients with the best prognostic profile (i.e., estrogen receptor α (ERα) positive and small-sized tumor without lymph node invasion) experience relapse in 10% to 20% of the cases at 5 years from surgery [3]. Traditional prognostic markers [1] are insufficient indicators of tumor aggressiveness and do not adequately discriminate among the different biological and clinical outcomes [3]. Therefore, new prognostic indicators are urgently needed.

Proteins that have roles in breast cancer growth, differentiation, invasion and/or metastasis can influence the biological progress of tumors, and can thus provide important prognostic information. One such candidate is Trop-2 [5]–[7]. Trop-1/Ep-CAM and Trop-2 [5]–[10] are monomeric trans-membrane glycoproteins that are expressed in human epithelial cells at diverse stages of differentiation [8], [9], [11]. Trop-1 and Trop-2 undergo homophylic binding, and are largely located at contact sites with adjacent cells, where they take part to the formation of specialized cell-cell adhesion structures [11], [12]. Over-expression of Trop-2 has been demonstrated to be necessary and sufficient to stimulate tumor growth [6]. Expression of Trop-1 and Trop-2 is associated with poor prognosis of several human cancers, including oral, pancreatic, gastric, ovarian, colorectal, breast and lung tumors [6], [13]–[15].

Trop-2 is synthesized in the endoplasmic reticulum, transported to, and glycosylated in the Golgi apparatus, and then sorted to the cell membrane [6]. The signaling function of Trop-2 [7] can be activated by antibody (Ab)-mediated cross-linking of cell-surface molecules [16] or by intra-membrane cleavage [17]. On the other hand, considerable amounts of Trop-2 are retained in intracellular compartments, in a broadly heterogeneous manner in different tumors [6], which suggests that this is part of the regulation of Trop-2 function. Here, we show that membrane localization and mature glycosylation of Trop-2 are associated with worse cancer patient survival, whereas Trop-2 intracellular retention is associated with less frequent disease relapse and better survival. These findings indicate that the Trop-2 activation state is a critical determinant of tumor progression, and they thus pave the way for their use of Trop-2 activations state as a novel prognostic indicators in breast cancer.

## Materials and Methods

### Patients

Informed written consent was obtained from all patients and the protocol of this study was approved by the University of Ferrara Research Ethics Committee and by the board of the Ministry of the University and Research (“Identification and validation of new markers of metastasizing phenotype of breast cancer”, prot. MM06095812_006, year 2000).

Seven hundred and two consecutive patients who underwent surgery for breast cancer between January 1989 and December 1993 at Ferrara University were analyzed. Patients were considered eligible according to the criteria listed in File S1: Patients and methods.

### Antibodies

The monoclonal anti-Trop-2 (m)Abs 162–46.2 (ATCC, HB-187) [18], 2EF and T16 [11] were purified from mouse ascites using protein-A Sepharose, as described previously [19]. They were used for flow cytometry (2EF, 162–46.2), confocal microscopy (T16, 2EF), Ab-mediated capping and electron microscopy (T16), and immunohistochemistry (162–46.2). The goat polyclonal anti-Trop-2 (p)Ab AF650 was obtained from R&D (R&D Systems, Inc. Minneapolis, MN), and was used for flow cytometry and immunohistochemistry.

### Trop-2 transport and internalization

Trop-2 transport and internalization were studied using flow cytometry, confocal microscopy, and electron microscopy, as detailed in File S1: Materials and methods.

### Association of Trop-2 with markers of tumor histotype and progression

Clusters of determinants of cancer aggressiveness were analyzed for a representative panel of breast cancer cell lines and control cancer cells (prostate, colon) using RT-PCR and flow cytometry, as detailed in File S1: Patients and methods.

### Immunohistochemistry

Formalin-fixed, paraffin-embedded (FFPE) breast tumor samples were obtained from mastectomies or excision biopsies. Tissue micro-array blocks were assembled, and the sections were analyzed as detailed in File S1: Patients and methods.

For Trop-2 (membrane and intracellular), Trop-1 and E-cadherin, total expression scores were obtained as described previously [20]. The total scores were computed as the product between the staining intensity scores (0, no reactivity; 1, weak staining; 2, moderate staining; 3, strong staining) and the percentage of positive cells scored (0, no stained cells; 1, 1–9%; 2, 10–49%; 3, 50–79%; 4, 80–100% stained cells). The total scores were then categorized as follows: 0, score 0; 1+, scores 1–4; 2+, scores 5–8; and 3+, scores 9–12. Expression levels were additionally categorized according to the percentage only of the stained cells, as: low, ≤5%; intermediate, 6–85%; high, ≥86%. Overall expression was further categorized as “+”, by grouping positive scores 1–12, or “-”, for score 0.

### Statistical analysis

To evaluate the associations between membrane and intracellular Trop-2 and the other clinico-pathological variables, adjusted odd ratios were estimated using multiple logistic regression. The Cohen's kappa statistic was used to quantify the agreement between membrane Trop-2 and intracellular (mAb- or pAb-detected) Trop-2. The R software (R Development Core Team. R: A language and environment for statistical computing. R Foundation for Statistical Computing, Vienna, Austria. 2011, www.R-project.org) was used throughout this study.

The effects of membrane and intracellular Trop-2 on patient outcome were evaluated according to distinct endpoints: (1) Hard endpoint: death from any cause (cumulative incidence, CI); (2) First failure: the occurrence of any first relapse over the follow-up period (recurrence, distant metastasis, contralateral tumor, other neoplasia, whichever occurred first after surgery; i.e., crude cumulative incidence, CCI). The CCI was obtained by taking into account death without evidence of disease as a competing risk [21]. The CI and CCI curves were estimated using the 1-Kaplan-Meier probability plots. The Cox proportional hazard regression model was used to assess the prognostic impact of Trop-2 in multivariate analysis. The effects of Trop-2 were adjusted for established prognostic factors; i.e., age, grading (G2–G3 *versus* G1), pathologic T stage (pT2-pT3 *versus* pT1), number of metastatic lymph nodes (1–3, 4–9 and >9 versus 0), ERα, HER-2/neu, p53, and E-cadherin expression levels. Adjusted curves for death CI for nil and positive scores of Trop-2 intracellular determination were determined according to the corrected group prognosis method using Cox regression [22].

The details of the statistical analysis are presented in File S1: Patients and methods.

## Results

### Cell-membrane Trop-2 signaling

**Figure 1:**
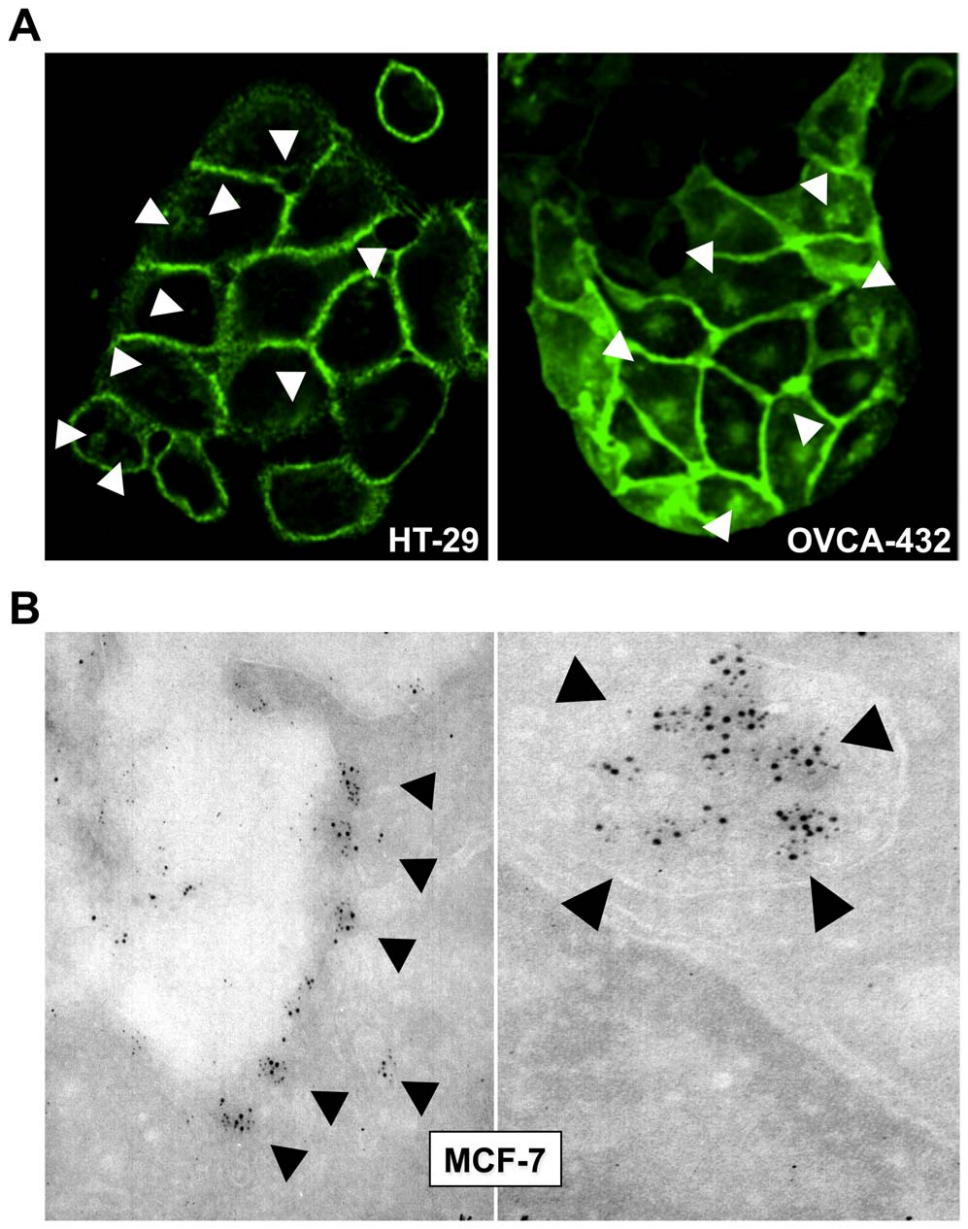
Cell membrane Trop-2 and internalization processes. Breast MCF-7, ovarian OVCA-432 and colon HT29 cancer cells were analyzed. (**A**) Cancer cell membrane *versus* intracytoplasmic Trop-2 retention. OVCA-432 and HT29 cells were stained with the T16 mAb. Arrrowheads indicate intracytoplasmic Trop-2 deposits. (**B**) Immuno-gold electron microscopy analysis of MCF-7 breast cancer cells. Trop-2 internalization was analyzed after induction of signaling (two-step cross-linking with the T16 mAb, followed by rabbit anti-mouse pAbs [16]). Black dots are gold nanospheres conjugated to anti-Trop-2 antibodies. (**left panel**) internalization of Trop-2 in intracellular, membrane-delimited areas; (**right panel**) endosome-like localization of internalized Trop-2.

Breast, ovary and colon cancer cells were assessed for their relative levels of cell membrane *versus* intracytoplasmic Trop-2 (Figure 1). The cognate Trop-1/Ep-CAM [10], [12] was used as an internal benchmark. Z-stack analysis allowed the identification of *bona fide* intracytoplasmic deposits *versus* membrane organelles; e.g., podosomes or macrovilli (Figure S1A). Distinct areas of localization of Trop-2 in intracellular granular deposits were shown for the majority of the cancer cells. Of note, most granules contained Trop-2, but not Trop-1 (Figure 1B), which indicates that Trop-1 and Trop-2 have differential retention mechanisms and distinct functional regulation.

At variance with the intracellular distribution profiles of Trop-1 and Trop-2 (Figure 1, Figure S1A), the membrane localization patterns of Trop-2 broadly overlapped with those of Trop-1 (Figure S1B), which in this case suggested parallel mechanisms of retention of Trop-1 and Trop-2 at the cell membrane. Association analysis showed corresponding patterns of Trop-1 and Trop-2 expression/localization at the cell membrane in tumors from patients (Table 1).

**Table 1:** Association between Trop-2 functional states and tumor pathobiological parameters.

Categorical variables^a^	*Overall* b	*Monoclonal* c			*Polyclonal* d		
	N (%)	low	high	*OR*	low	high	*OR*
		(score = 0)	(score>0)		(score = 0)	(score>0)	
		N (%)	N (%)		N (%)	N (%)	
*Age (n_M_ = 620, n_P_ = 617, P_M_ = 0.98, P_P_ = 0.75)*							
34–40	51 (7.3)	11 (6.7)	35 (7.7)	1	10 (7.5)	37 (7.6)	1
41–50	144 (20.5)	36 (21.8)	89 (19.6)	0.82	29 (21.8)	102 (21.1)	0.87
51–55	87 (12.4)	20 (12.1)	56 (12.3)	0.99	11 (8.3)	67 (13.8)	0.75
56–70	274 (39.0)	67 (40.6)	179 (39.3)	0.97	57 (42.9)	184 (38.0)	0.59
71–90	146 (20.8)	31 (18.8)	96 (21.1)	0.91	26 (19.5)	94 (19.4)	0.71
*Histotype (n_M_ = 620, n_P_ = 617, P_M_ = 0.25, P_P_ = 0.04)*							
Ductal	527 (75.1)	109 (66.1)	358 (78.7)	1	104 (78.2)	364 (75.2)	1
Lobular	109 (15.5)	35 (21.2)	61 (13.4)	0.64	13 (9.8)	79 (16.3)	2.92
other histotypes	66 (9.4)	21 (12.7)	36 (7.9)	0.56	16 (12.0)	41 (8.5)	0.89
*pT (n_M_ = 618, _nP = _615, P_M_ = 0.03, P_P_ = 0.61)*							
pT1	451 (64.4)	103 (62.8)	296 (65.2)	1	78 (58.6)	316 (65.5)	1
pT2	236 (33.7)	59 (36.0)	147 (32.4)	0.51	52 (39.1)	156 (32.4)	0.77
pT3	13 (1.9)	2 (1.2)	11 (2.4)	1.23	3 (2.3)	10 (2.1)	0.55
*Histological grade (n_M_ = 619, n_P_ = 616, P_M_ = 0.08, P_P_ = 0.45)*							
G1	135 (19.3)	41 (24.8)	71 (15.6)	1	23 (17.3)	93 (19.3)	1
G2	427 (61.0)	107 (64.8)	276 (60.8)	1.3	76 (57.1)	299 (61.9)	0.79
G3	138 (19.7)	17 (10.3)	107 (23.6)	2.67	34 (25.6)	91 (18.8)	0.57
*Nodal status (n_M_ = 620, n_P_ = 617, P_M_ = 0.51, P_P_ = 0.04)*							
negative	393 (56.0)	93 (56.4)	253 (55.6)	1	81 (60.9)	257 (53.1)	1
positive	309 (44.0)	72 (43.6)	202 (44.4)	0.85	52 (39.1)	227 (46.9)	1.77
*Estrogen receptor α(n_M_ = 512, n_P_ = 511, P_M_ = 0.80, P_P_ = 0.06)*							
≤10%	127 (21.5)	22 (16.1)	85 (22.7)	1	29 (27.4)	85 (21.0)	1
>10%	463 (78.5)	115 (83.9)	290 (77.3)	1.1	77 (72.6)	320 (79.0)	2.14
*Progesterone receptor (n_M_ = 507, n_P_ = 511, P_M_ = 0.61, P_P_ = 0.22)*							
≤10%	173 (29.6)	37 (27.2)	111 (29.9)	1	29 (27.9)	120 (29.9)	1
>10%	412 (70.4)	99 (72.8)	260 (70.1)	1.18	75 (72.1)	282 (70.1)	0.63
*HER-2/neu (n_M_ = 611, n_P_ = 604, P_M_ = 0.09, P_P_ = 0.87)*							
≤10%	470 (69.5)	130 (79.8)	291 (65.0)	1	91 (70.0)	322 (67.9)	1
>10%	206 (30.5)	33 (20.2)	157 (35.0)	1.66	39 (30.0)	152 (32.1)	0.95
*p53 (n_M_ = 601, n_P_ = 593, P_M_ = 0.39, P_P_ = 0.92)*							
≤10%	308 (46.7)	85 (55.2)	196 (43.8)	1	58 (44.3)	220 (47.6)	1
>10%	352 (53.3)	69 (44.8)	251 (56.2)	1.23	73 (55.7)	242 (52.4)	1.03
*Membrane Trop-1 (n_M_ = 603, n_P_ = 580, P_M_ = 0.02, P_P_ = 0.004)*							
low	428 (66.7)	122 (77.2)	271 (60.9)	1	95 (79.2)	293 (63.7)	1
high	214 (33.3)	36 (22.8)	174 (39.1)	1.9	25 (20.8)	167 (36.3)	2.43
*Membrane Trop-2 (n_M_ = 604, n_P_ = 617, P_M_ = 0.59, P_P_ = 0.10)*							
low	149 (22.4)	39 (25.7)	88 (19.5)	1	39 (29.3)	90 (18.6)	1
high	516 (77.6)	113 (74.3)	364 (80.5)	1.17	94 (70.7)	394 (81.4)	1.65
*E-cadherin (n_M_ = 574, n_P_ = 588, P_M_ = 0.03, P_P_ = 0.08)*							
low	304 (48.4)	70 (47.6)	200 (46.8)	1	75 (59.1)	208 (45.1)	1
high	324 (51.6)	77 (52.4)	227 (53.2)	0.55	52 (40.9)	253 (54.9)	1.61
*Adjuvant therapies (n_M_ = 503, n_P_ = 505)*							
No therapy	239 (41.6)	46 (34.1)	154 (41.8)		47 (44.8)	164 (41.1)	
Chemotherapy (CT)	99 (17.2)	25 (18.5)	65 (17.7)		20 (19.0)	66 (16.5)	
Hormone therapy (HT)	205 (35.8)	59 (43.7)	131 (35.6)		35 (33.3)	141 (36.2)	
CT plus HT	31 (5.4)	5 (3.7)	18 (4.9)		3 (2.9)	25 (6.2)	

a : variables analyzed.

b : overall distribution for each of the variables included in the study.

c : distribution of scores of cytoplasmic Trop-2 expression (low or high).

d : distribution of scores of membrane Trop-2 expression (low or high). Both raw (N) and relative (%) frequencies are reported. OR: adjusted odds ratios for intracellular Trop-2 overexpression (high *versus* low), obtained by multiple logistic regression; categories used as reference have OR = 1. pT: pathological stage. Wald test P values for the mAb (P_M_) and for the pAb (P_P_) scores and associated numbers of cases analyzed (*n_M_, n_P_*) are indicated.

**Figure 2:**
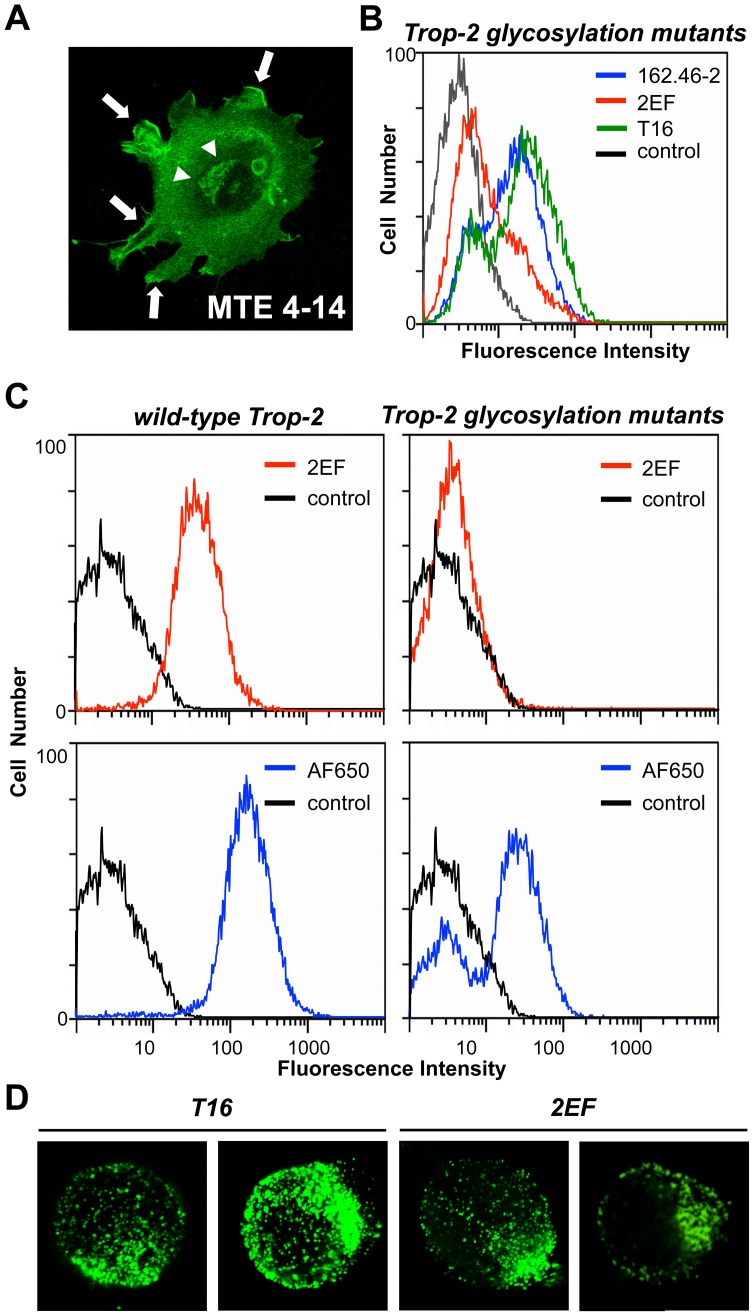
Glycosylation-dependent Trop-2 transport and signaling. (**A**) Binding of 2EF to fully-matured forms of Trop-2. Ample binding to the cell membrane (arrows) was revealed. Strong staining of the Golgi apparatus was also shown (arrowheads), consistent with recognition of glycosylated Trop-2. (**B**) Flow cytometry analysis of KM12SM cells stably transfected with glycosylation mutants. Living cells were analyzed for membrane-only staining. 2EF, T16 and 162–46.2: unconjugated anti-Trop-2 mAbs, followed by rabbit anti-mouse Alexa-488; control: irrelevant antibody stained cells. (**C**) Flow cytometry analysis of KM12SM stably transfected with wild-type Trop-2 or deglycosylated variant. Living cells were analyzed for membrane-only staining. 2EF: anti-Trop-2 Alexa-488 conjugated mAb; AF650: anti-Trop-2 goat pAb; control: irrelevant antibody-stained cells. Living cells were analyzed for membrane-only staining. (**D**) MTE 4–14 cells transfected with Trop-2 subjected to Ab-mediated capping. The T16 (left) and 2EF (right) mAbs were used for primary Ab incubation, followed by cross-linking with a secondary Ab conjugated with Alexa488.

After synthesis in the endoplasmic reticulum, membrane proteins can be subjected to N-glycosylation in the Golgi apparatus prior to subsequent sorting to the cell membrane. As the extracellular domain of Trop-2 contains four putative N-glycosylation sites [5], we generated an entirely deglycosylated Trop-2 variant through site-directed mutagenesis of the N-glycosylation sites to Ala (manuscript in preparation), and expressed this Trop-2 variant in colon cancer cells. We also developed a novel mAb (2EF) directed against the Trop-2 extracellular domain, which specifically recognizes glycosylated forms of Trop-2. Quantitative flow cytometry revealed that the 2EF mAb indeed fails to bind deglycosylated Trop-2 (Figure 2B, C), at variance with the mAbs 162–46.2 and T16 and the pAb AF650 which recognize both fully glycosylated and deglycosylated Trop-2 (Figure 2B, C). The 2EF mAb was also used for immunofluorescence analysis of Trop-2-expressing cells (Figure 2A). Here 2EF showed localization of glycosylated Trop-2 in intracellular deposits (Figure 2A, arrowheads), in agreement with our previous observations [6]. Formal proof that membrane molecules are functionally competent was then sought. First, we showed that fully glycosylated membrane Trop-2 molecules can be internalized for degradation/recycling. Antibody-mediated cross-linking of the membrane-associated Trop-2 revealed capping of the Ab/Trop-2 complex, followed by internalization in intracellular deposits (Movie S1, Figure 2D). Furthermore, fully glycosylated molecules cross-linked in vivo by 2EF were shown to be functionally competent and to induce signaling (manuscript in preparation). Thus, intracellular Trop-2 is a candidate for a signaling-inactive form of Golgi-residing intermediates of translocation to the cell membrane or internalized deposits of recycled/degraded molecules. Hence, membrane and intracellular Trop-2 may have different impact on tumor prognosis.

### Association of membrane Trop-2 with tumor progression determinants in patients

An association of Trop-2 with cancer progression determinants was found *in vitro* (Supporting online results and Table S1 in File S1). The association of Trop-2 with determinants of tumor aggressiveness was then explored in cancer patients. The mAb 162–46.2 (from now on called ‘mAb’) was found to specifically detect the cytoplasmic pool of Trop-2 in FFPE samples, whereas the pAb AF650 (from now on called ‘pAb’) detected both membrane- and cytoplasm-associated Trop-2. Hence, both antibodies were used for discrimination of membrane and cytoplasm-associated Trop-2 in human tissues, to assess the impact of these two pools on the prognosis of breast cancer patients. A consecutive breast cancer case series from a single institution (702 cases; 8 years median follow-up) was analyzed (Table 1). Distinct methodologies, including alternative categorization procedures (immunohistochemistry score, percent of positive cell classes), were used for analysis of patient data, to dissect out their relative impact on patient prognosis (for further details, see Supporting Materials and Methods section in File S1). There was a significant association of mAb-detected Trop-2 with pathological stage (P = 0.04) and E-cadherin levels (P = 0.04). On the other hand, the pAb-detected Trop-2 was significantly associated with nodal status (P = 0.04) and histotype (P = 0.04). Intracellular Trop-2, as detected by both the mAb and pAb, was associated with the membrane-localized Trop-1, but not with the membrane-associated Trop-2, indicating that membrane *versus* intracellularly-retained Trop-2 are distinct functional variables. Consistent with this, the k-statistic for agreement between the membrane and intracellular Trop-2 (mAb) was low (0.065; confidence interval: 0.017–0.148), as it was also low that for mAb-detected versus pAb-detected intracellular Trop-2 (0.112; confidence interval: 0.025–0.200).

### Multiple correspondence association and principal component analysis

These findings led us to further explore the association of the membrane and intracytoplasmic Trop-2 with aggressiveness determinants by multiparametric multiple correspondence association (Figure S2A). The horizontal (first) axis mainly separated low ERα/G3/high HER-2 (left) from high ERα/G1/low HER-2 and “other histotypes” (right). The vertical axis mainly separated high Trop-1/high E-cadherin (top) from low Trop-1/low E-cadherin/low membrane-associated Trop-2. The mAb-detected intracellular Trop-2 nil score is positioned near favourable prognostic factors.

The Trop-2 association with tumor aggressiveness determinants was then investigated using principal component analysis (Figure S2B). ERα and progesterone receptor (PgR) showed high direct correlation with each other, and high inverse correlation with HER-2/neu and p53. E-cadherin, membrane-associated Trop-1, membrane-associated Trop-2 and pAb-detected intracellular Trop-2 were intercorrelated, but were not correlated with the other markers. On the other hand, the mAb-detected intracellular Trop-2 showed low correlation with all of the other bio-markers analyzed, which suggests that it has potential for high discriminating power as an independent variable.

### Mature versus immature Trop-2 forms in breast cancer

**Figure 3:**
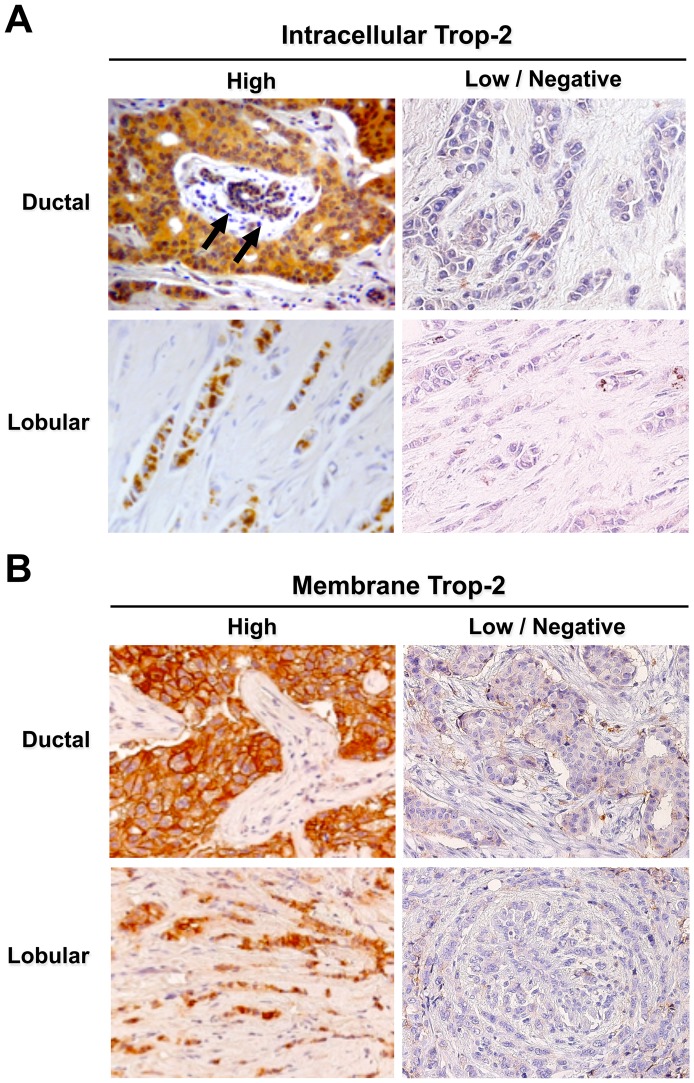
Immunohistochemistry analysis of Trop-2 expression in breast cancer. Breast cancer samples were analyzed by immunohistochemistry using the 162–46.2 anti-Trop-2 mAb [32] for detection of the intracellular Trop-2 (**A**) and with the R&D AF650 goat pAb for detection of membrane-associated Trop-2 (**B**). Images are representative cases of ductal (top panels) and lobular (bottom panels) cancers. Arrows: normal breast ducts. Expression levels were classified as high and low/negative. Magnification is 40x.

High expression of membrane-associated Trop-2 was observed in 77.6% of the cancers analyzed. High levels of intracellular Trop-2 were detected in 73.4% of cases using the anti-Trop-2 mAb, and 78.4% using the pAb (Table 1 and Tables S2, S3 in File S1). The highest intensity of Trop-2 expression was observed in ductal carcinomas, with lower levels in lobular tumors and “other histotype” cases. In ductal and lobular breast cancers, the anti-Trop-2 mAb mostly stained intracellular compartments, and granular staining patterns were frequently observed (Figure 3A). Previous data indicated that these regions correspond to the endoplasmic reticulum, the Golgi apparatus and post-Golgi compartments, including early endosomes, late endosomes and intracytoplasmic storage vesicles [6], [23].

Immunohistochemistry staining patterns of pAb-detected Trop-2 are shown in Figure 3B. The highest reactivity was found against cancer cell membranes. Fainter, mostly homogeneous reactivity was observed intracytoplasmically in a distinct fraction of membrane-reactive cells. Heterogeneity of expression patterns of Trop-2 was found in essentially all breast tumor histotypes.

### Impact of Trop-2 functional states on patients survival

Taken together, our findings suggest an important impact of Trop-2 functional states on patient survival. Hence, we followed our breast cancer case series for 96 months. During this time, for the first events that developed, 110 patients showed distant metastases (CCI, 15.7% [13.1–18.5%]), 52 a local relapse (CCI, 7.6% [5.8–9.7%]), 14 a contra-lateral tumor (CCI, 2.0% [1.2–3.2%]) and 33 other malignancies (CCI, 4.8% [3.4–6.5%]). Death occurred in 96 cases (CCI, 14.2% [11.8–16.9%]). The absolute frequencies of the first adverse events during follow-up were analyzed according to lymph node status (Table S4 in File S1), expression of immature intracellular Trop-2 (Table S5 in File S1), mature intracellular Trop-2 (Table S6 in File S1), and membrane Trop-2 (Table S7 in File S1).

**Figure 4:**
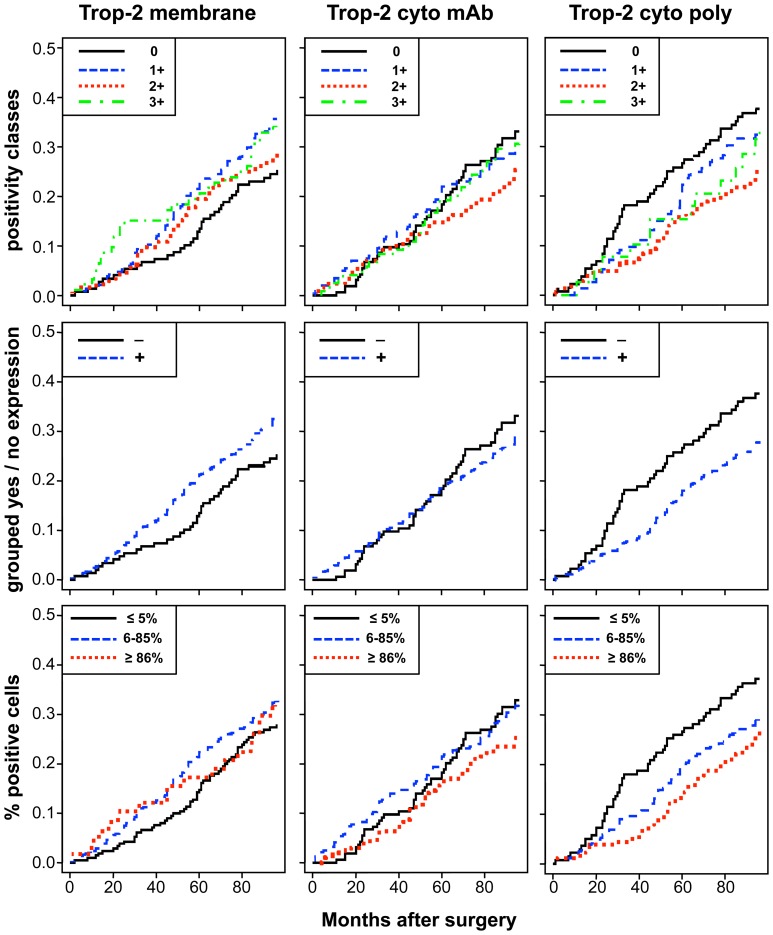
Impact of membrane *versus* intracellular Trop-2 on patient survival. Cumulative incidence (CI) estimates of death from any cause were obtained as 1-Kaplan-Meier curves for distinct Trop-2 expression sub-groups (cell membrane; mAb-detected intracellular; pAb-detected intracellular). Trop-2 expression was categorized according to (**top**) intensity scores (0, 1+, 2+, 3+), (**middle**) intensity grouping, i.e. positive scores 1–12 (+) versus score 0 (−), (**bottom**) percentage of stained cells (low, ≤5%; intermediate, 6–85%; high, ≥86%), as indicated in the panels.

**Figure 5:**
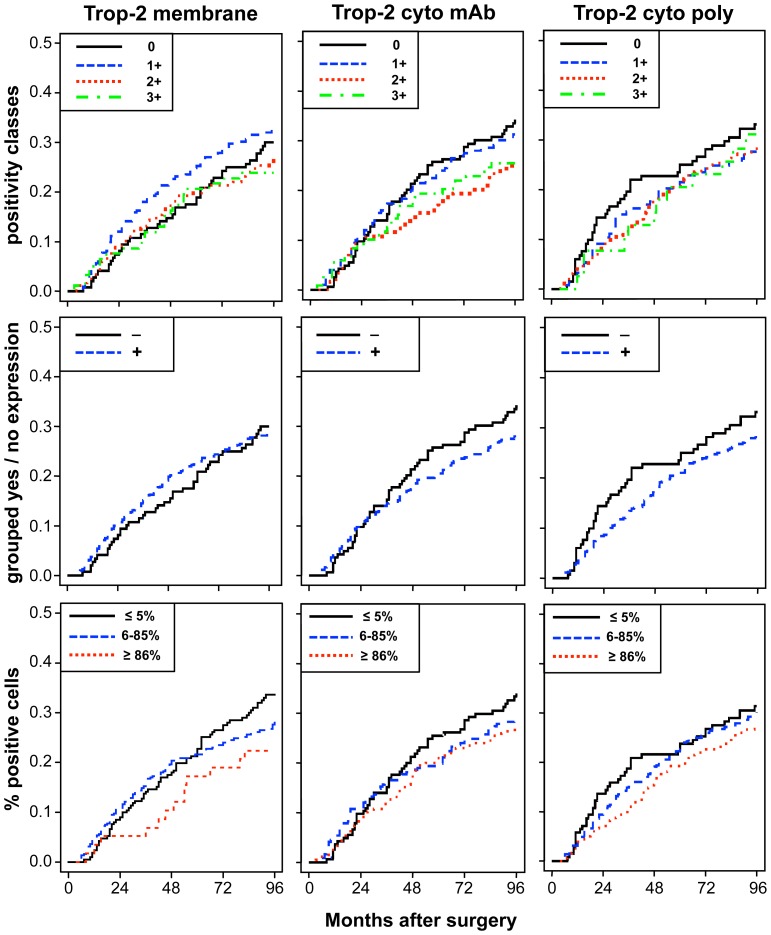
Impact of membrane versus intracellular Trop-2 on disease relapse. Crude cumulative incidence (CCI) estimates of disease relapse were obtained as 1-Kaplan-Meier curves for distinct Trop-2 expression sub-groups (cell membrane; mAb-detected intracellular; pAb-detected intracellular). Trop-2 expression was categorized according to (**top**) intensity scores (0, 1+, 2+, 3+), (**middle**) intensity grouping, i.e. positive scores 1–12 (+) versus score 0 (−), (**bottom**) percentage of stained cells (low, ≤5%; intermediate, 6–85%; high, ≥86%), as indicated in the panels. CCI were estimated accounting for death as a competing risk.

The unadjusted estimates of death CI and of relapse CCI according to different levels of membrane and intracellular Trop-2 are shown in Figures 4 and 5. Table 2 shows the results of univariate and multiple Cox regression models, to estimate unadjusted and adjusted hazard ratios of positive *versus* nil scores. Membrane Trop-2 appeared as an unfavorable prognostic risk factor [24]. Considering death CI, the adjusted hazard ratio for scores 1+ *versus* 0 was 1.63 (P = 0.04). A similar trend was observed for Trop-2 expression scores 1+2+3+ *versus* 0 (Table 2).

**Table 2:** Proportional hazard Cox regression analysis.

	Hazard ratio			
	Cumulative incidence		CCI of relapse^a^	
	Unadjusted	Adjusted	Unadjusted	Adjusted
***Membrane Trop-2***				
1+ *versus* 0	**1.50 (0.04)**	**1.63 (0.04)**	1.22 (0.30)	1.17 (0.49)
2+ *versus* 0	1.17 (0.47)	0.99 (0.97)	0.92 (0.68)	0.80 (0.38)
3+ *versus* 0	1.46 (0.12)	1.19 (0.58)	0.85 (0.55)	0.90 (0.74)
123+ *versus* 0	**1.37 (0.08)**	1.30 (0.23)	1.04 (0.82)	0.98 (0.93)
intermediate *versus* low	1.23 (0.19)	1.13 (0.52)	0.88 (0.40)	0.88 (0.50)
high *versus* low	1.16 (0.58)	1.45 (0.24)	0.67 (0.18)	0.80 (0.51)
***Intracellular Trop-2 (mAb)***				
1+ *versus* 0	0.91 (0.62)	0.80 (0.32)	0.93 (0.69)	0.82 (0.37)
2+ *versus* 0	0.73 (0.17)	**0.55 (0.03)**	0.70 (0.11)	**0.53 (0.02)**
3+ *versus* 0	0.95 (0.80)	0.70 (0.18)	0.72 (0.15)	**0.56 (0.03)**
123+ *versus* 0	0.87 (0.39)	**0.69 (0.05)**	0.81 (0.18)	**0.67 (0.04)**
intermediate *versus* low	0.99 (0.93)	0.91 (0.66)	0.87 (0.44)	0.85 (0.42)
high *versus* low	0.75 (0.15)	**0.48 (0.003)**	0.75 (0.14)	**0.51 (0.004)**
***Intracellular Trop-2 (pAb)***				
1+ *versus* 0	0.80 (0.27)	1.16 (0.56)	0.78 (0.25)	0.98 (0.93)
2+ *versus* 0	**0.59 (0.004)**	**0.59 (0.03)**	0.79 (0.20)	0.76 (0.24)
3+ *versus* 0	0.81 (0.50)	0.88 (0.75)	0.84 (0.59)	0.82 (0.67)
123+ *versus* 0	**0.67 (0.02)**	**0. 70 (0.08)**	0.79 (0.17)	0.77 (0.21)
intermediate *versus* low	**0.72 (0.07)**	0.76 (0.21)	0.90 (0.59)	0.85 (0.46)
high *versus* low	**0.62 (0.02)**	**0.55 (0.02)**	0.77 (0.20)	0.73 (0.20)

Unadjusted and adjusted hazard ratios according to Trop-2 expression levels (at the cell membrane or intracellular, as detected by mAb or pAb) and corresponding P values.

a : cause-specific hazard ratios. The adjusted models included age (continuous linear), grading, pT stage (pT2+pT3 versus pT1), number of positive lymph nodes (0, 1–3, 4–9, >9), ERα, HER-2/neu, p53 and E-cadherin (cut-off: 10% positive cells). Low; ≤5% positive cells; intermediate, 6–85%; high, ≥86%. Significantly different values and trends are in bold.

Both mature and immature intracellular Trop-2 had a favorable prognostic impact on death CI. The adjusted hazard ratio for scores 1+, 2+, 3+ *versus* score 0, for death from any cause was 0.69 (P = 0.05) for mAb, and 0.70 (P = 0.08) for pAb determination. Remarkably, the adjusted hazard ratio for high *versus* low expression of intracellular mAb-detected Trop-2 on death from any cause was 0.48 (P = 0.003), whereas that for intracellular pAb-detected Trop-2 was 0.55 (P = 0.02) (Figure 4). Multivariable adjustment increased the statistical significance of the Trop-2 scores for mAb determination, while maintaining that for the Trop-2 pAb scores (Figure S3).

There were corresponding impacts on disease relapse, with a hazard ratio of 0.67 (P = 0.04) for the mAb determination of intracellular Trop-2. The adjusted hazard ratio for the intensity of high *versus* low expression of intracellular Trop-2 (mAb) on first relapse was 0.51 (P = 0.004) (Figure 5). The prognostic impact of intracellular Trop-2 expression on patient outcome, as assessed by mAb staining, markedly improved after adjusting for other prognostic factors, supporting a key role for Trop-2 as a prognostic determinant of overall survival and disease relapse.

## Discussion

Trop-2 is a key driver of growth of transformed cells, whether through over-expression of growth-driving wild-type Trop-2 [6], or through the generation of oncogenic bi-cistronic Cyclin D1-TROP2 mRNA chimeras [25]. On the other hand, our findings show that large amounts of Trop-2 are retained in intracellular compartments in a widely heterogeneous manner in tumors; e.g., in breast, ovary and colon cancers. This was at variance with the cognate Trop-1/Ep-CAM, which suggested distinct regulation of Trop-2 function. A glycosylation-dependent anti-Trop-2 mAb was developed to specifically assess the signaling competence of post-translationally modified Trop-2. Using a case series of breast cancer patients built following the REMARK recommendations for tumor marker prognostic studies [22] (Table S8 in File S1), membrane Trop-2 was shown to be associated with major determinants of biological history of breast cancer, i.e. membrane Trop-1 and CD44v, with ERα/PgR-negative cases, and with distinct breast cancer subgroups (luminal, triple negative).

These findings suggested a deep impact of Trop-2 functional state on breast cancer biological history. Membrane-associated Trop-2 was found to have an unfavorable prognostic impact on patient survival [24]. On the other side, intracellular Trop-2 showed a deep, positive impact on both patient survival and disease recurrence. Taken together, our findings identify Trop-2 as a key determinant of patient survival, opening novel avenues of research on the pathways that drive tumor progression.

Current predictors of overall survival are tumor size, grading, fraction of proliferating cells, and vascular invasion [26], inevitably linking tumor prognostic determination to late-comer indicators. On the other hand, molecular markers like p53, HER-2/neu, and ERα [27], show little impact on patient survival [26]. Immunohistochemistry markers of favorable prognosis of breast cancer include only ERα, PgR [27], [28], Bcl-2 [29] and E-cadherin [20]. Further, hormone receptors are weak predictors of patient outcome [26]–[28], [30]; only the combined absence of ERα, PgR and HER-2/neu associates with aggressive triple-negative breast cancers [31]. Bcl-2 antagonizes the induction of tumor cell apoptosis, but it is also associated with tumor differentiation and longer disease-free survival [29]. Loss of E-cadherin ([20] and references therein), or functional inactivation of this adhesion molecule (manuscript in preparation) are required for invasion and metastasis. However, favorable *versus* unfavorable prognostic impacts of E-cadherin depend on its expression levels [20]. Both higher-than-normal and lower-than-normal E-cadherin expression levels are associated with worse prognosis [20], thus posing limits to the use of E-cadherin as a dichotomous prognostic marker.

On the other hand, our findings indicate that the states of Trop-2 can serve as a powerful, differential indicator of sharply distinct disease outcome, thus paving the way for their use for identifying patient subgroups with distinct cancer-associated risk.

## Supporting Information

Figure S1 **Trop-2 cell membrane** ***versus*** **intracytoplasmic retention.**
(TIF)

Figure S2 **Association analysis for membrane and intracellular Trop-2.**
(TIF)

Figure S3 **Adjusted impact on outcome for membrane and intracellular Trop-2.**
(TIF)

Movie S1 **Trop-2 capping by antibodies cross-linking.**
(MOV)

File S1 **This file contains Supporting Materials and Methods, Supporting Results, Supporting References, and Tables S1-S8.** Table S1, Association between Trop-2 surface expression and tumor progression markers. Table S2, Frequency of tumor histotypes. Table S3, Intensity scores distribution. Table S4, Absolute frequency of first adverse events by lymph node status. Table S5, Absolute frequency of first adverse events by percentage of cells stained for intracellular Trop-2 – mAb detection. Table S6, Absolute frequency of first adverse events within 96 months after surgery by percentage of cells stained for intracellular Trop-2 – polyclonal antibody detection. Table S7, Absolute frequency of first adverse events by percentage of cells stained for membrane Trop-2. Table S8, Adherence to REMARK criteria (adapted from [23] in Supporting References).
(DOC)
