# Correction: Trop-2 Is a Determinant of Breast Cancer Survival

**DOI:** 10.1371/journal.pone.0110606

**Published:** 2014-10-10

**Authors:** The PLOS ONE Staff

There is an error in the third sentence of the second paragraph of the “Statistical Analysis” subsection of the Materials and Methods. The correct sentence is: The CI curves were estimated using the 1-Kaplan-Meier probability plots.

There are a number of errors in the headings for Table 2. Please see the corrected Table 2 here.

**Table 2:** Proportional hazard Cox regression analysis.

	Hazard ratio			
	Death		Relapse a	
	Unadjusted	Adjusted	Unadjusted	Adjusted
***Membrane Trop-2***				
1+ *versus* 0	**1.50 (0.04)**	**1.63 (0.04)**	1.22 (0.30)	1.17 (0.49)
2+ *versus* 0	1.17 (0.47)	0.99 (0.97)	0.92 (0.68)	0.80 (0.38)
3+ *versus* 0	1.46 (0.12)	1.19 (0.58)	0.85 (0.55)	0.90 (0.74)
123+ *versus* 0	**1.37 (0.08)**	1.30 (0.23)	1.04 (0.82)	0.98 (0.93)
intermediate *versus* low	1.23 (0.19)	1.13 (0.52)	0.88 (0.40)	0.88 (0.50)
high *versus* low	1.16 (0.58)	1.45 (0.24)	0.67 (0.18)	0.80 (0.51)
***Intracellular Trop-2 (mAb)***				
1+ *versus* 0	0.91 (0.62)	0.80 (0.32)	0.93 (0.69)	0.82 (0.37)
2+ *versus* 0	0.73 (0.17)	**0.55 (0.03)**	0.70 (0.11)	**0.53 (0.02)**
3+ *versus* 0	0.95 (0.80)	0.70 (0.18)	0.72 (0.15)	**0.56 (0.03)**
123+ *versus* 0	0.87 (0.39)	**0.69 (0.05)**	0.81 (0.18)	**0.67 (0.04)**
intermediate *versus* low	0.99 (0.93)	0.91 (0.66)	0.87 (0.44)	0.85 (0.42)
high *versus* low	0.75 (0.15)	**0.48 (0.003)**	0.75 (0.14)	**0.51 (0.004)**
***Intracellular Trop-2 (pAb)***				
1+ *versus* 0	0.80 (0.27)	1.16 (0.56)	0.78 (0.25)	0.98 (0.93)
2+ *versus* 0	**0.59 (0.004)**	**0.59 (0.03)**	0.79 (0.20)	0.76 (0.24)
3+ *versus* 0	0.81 (0.50)	0.88 (0.75)	0.84 (0.59)	0.82 (0.67)
123+ *versus* 0	**0.67 (0.02)**	**0. 70 (0.08)**	0.79 (0.17)	0.77 (0.21)
intermediate *versus* low	**0.72 (0.07)**	0.76 (0.21)	0.90 (0.59)	0.85 (0.46)
high *versus* low	**0.62 (0.02)**	**0.55 (0.02)**	0.77 (0.20)	0.73 (0.20)

Unadjusted and adjusted hazard ratios according to Trop-2 expression levels (at the cell membrane or intracellular, as detected by mAb or pAb) and corresponding P values.

a : cause-specific hazard ratios. The adjusted models included age (continuous linear), grading, pT stage (pT2+pT3 versus pT1), number of positive lymph nodes (0, 1-3, 4-9, >9), ERα, HER-2/neu, p53 and E-cadherin (cut-off: 10% positive cells). Low; ≤ 5% positive cells; intermediate, 6-85%; high, ≥ 86%. Significantly different values and trends are in bold.

There is an error in the legend for Figure 4. Please see the corrected Figure 4 here.

**Figure 4:**
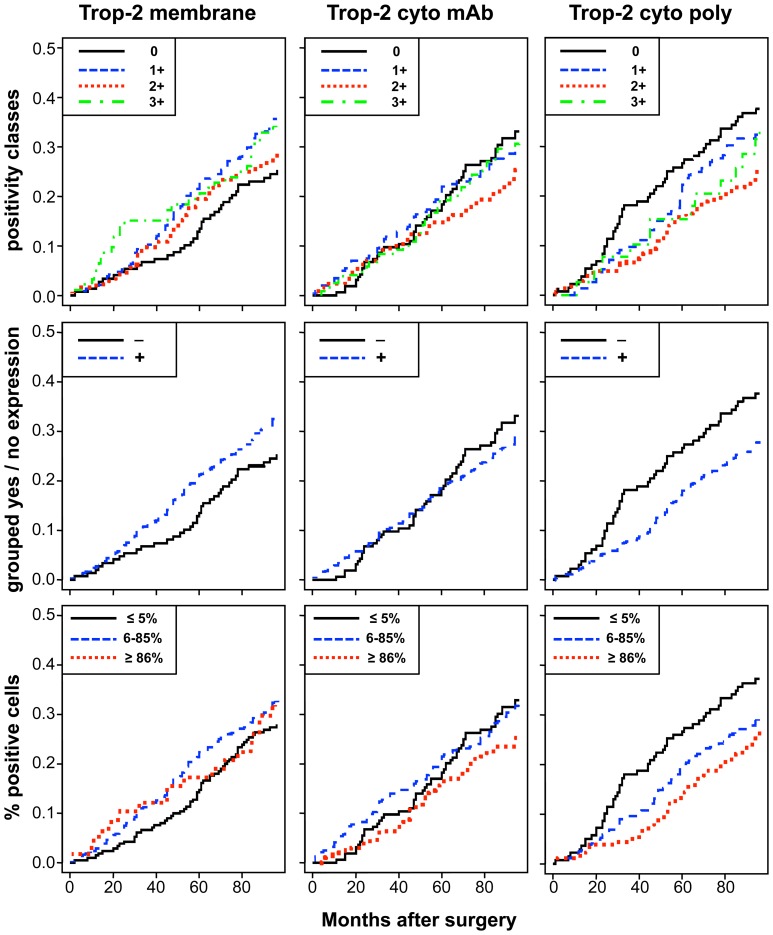
Impact of membrane *versus* intracellular Trop-2 on patient survival. Cumulative incidence (CI) estimates of death from any cause are reported on the y-axis as a function of follow-up time for distinct Trop-2 expression sub-groups (cell membrane; mAb-detected intracellular; pAb-detected intracellular). Trop-2 expression was categorized according to (**top**) intensity scores (0, 1+, 2+, 3+), (**middle**) intensity grouping, i.e. positive scores 1–12 (+) versus score 0 (−), (**bottom**) percentage of stained cells (low, ≤5%; intermediate, 6–85%; high, ≥86%), as indicated in the panels.

There is an error in the legend for Figure 5. Please see the corrected Figure 5 here.

**Figure 5:**
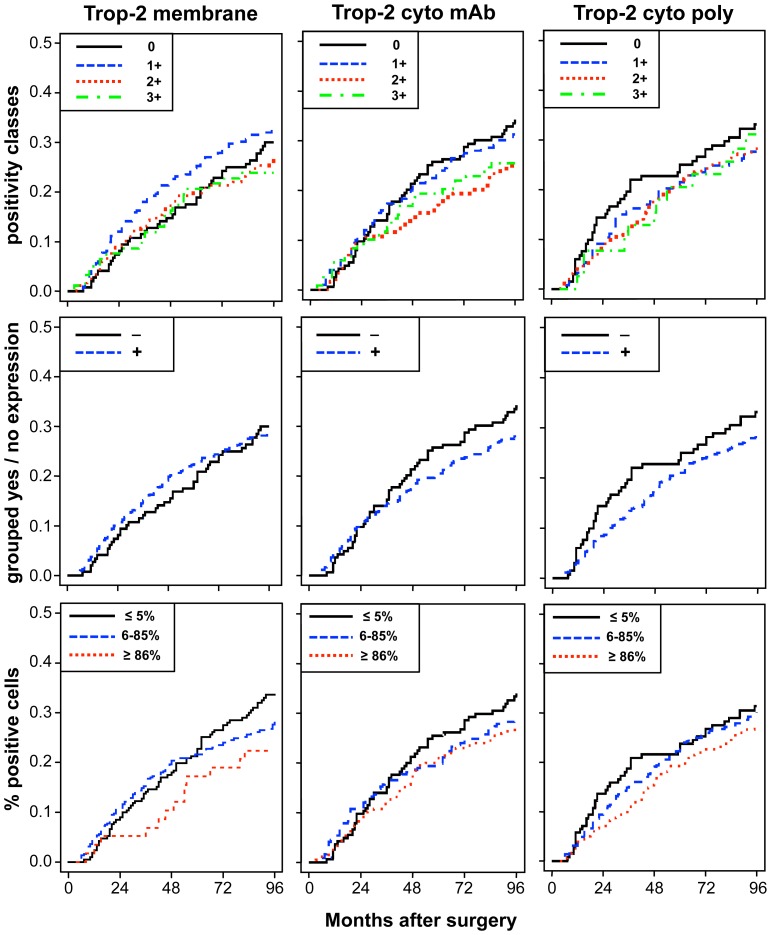
Impact of membrane versus intracellular Trop-2 on disease relapse. Crude cumulative incidence (CCI) estimates of disease relapse are reported on the y-axis as a function of follow-up time for distinct Trop-2 expression sub-groups (cell membrane; mAb-detected intracellular; pAb-detected intracellular). Trop-2 expression was categorized according to (**top**) intensity scores (0, 1+, 2+, 3+), (**middle**) intensity grouping, i.e. positive scores 1–12 (+) versus score 0 (−), (**bottom**) percentage of stained cells (low, ≤5%; intermediate, 6–85%; high, ≥86%), as indicated in the panels. CCI were estimated accounting for death as a competing risk.
