# Supplementary figures and images for: Trop-2 Is a Determinant of Breast Cancer Survival
Source: PLoS One. 2014 May 13;9(5):e96993. doi: 10.1371/journal.pone.0096993 (PMC4019539; doi:10.1371/journal.pone.0096993)

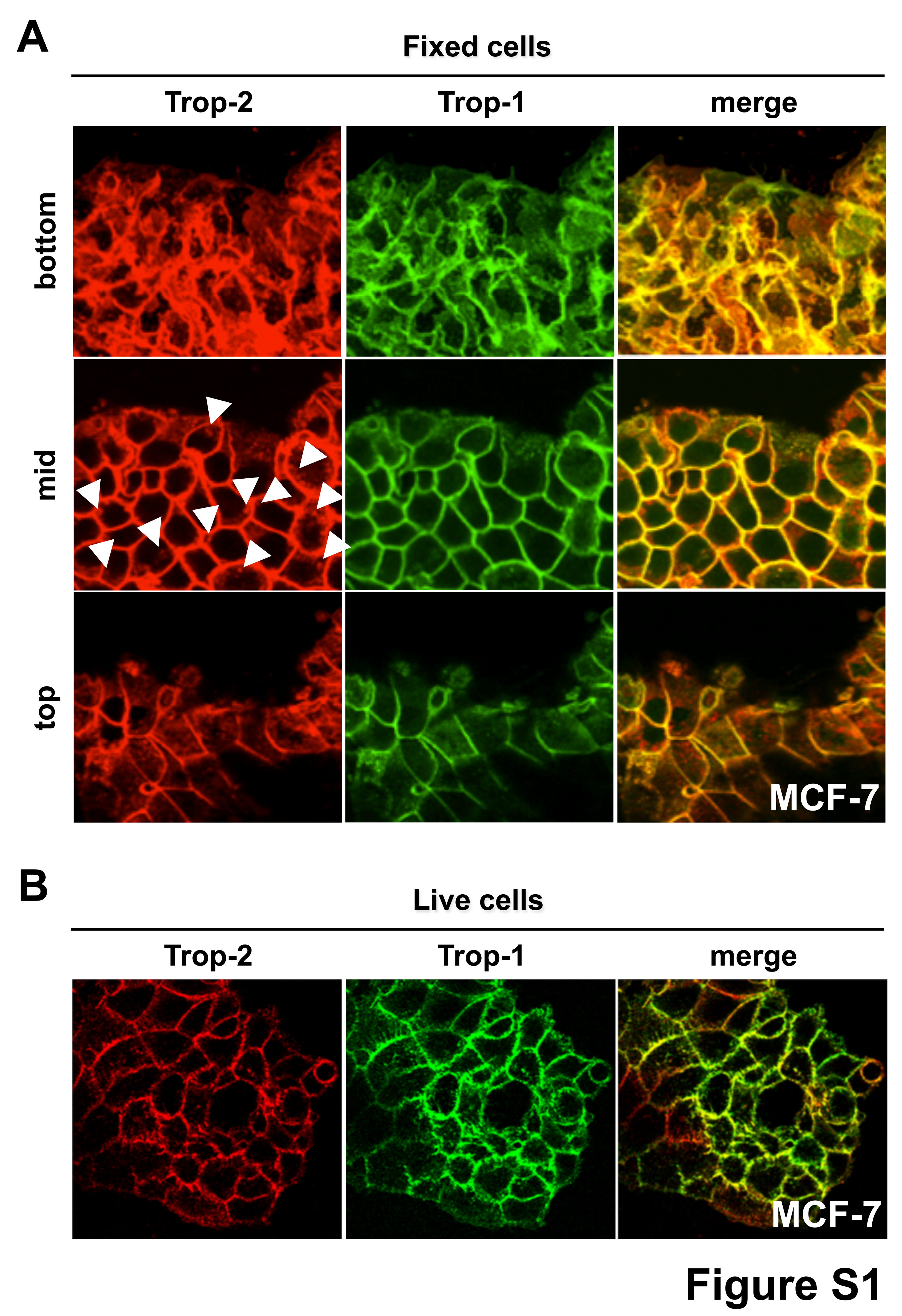

Supplement: Figure S1 — Trop-2 cell membrane versus intracytoplasmic retention. (TIF) [file pone.0096993.s001.tif]

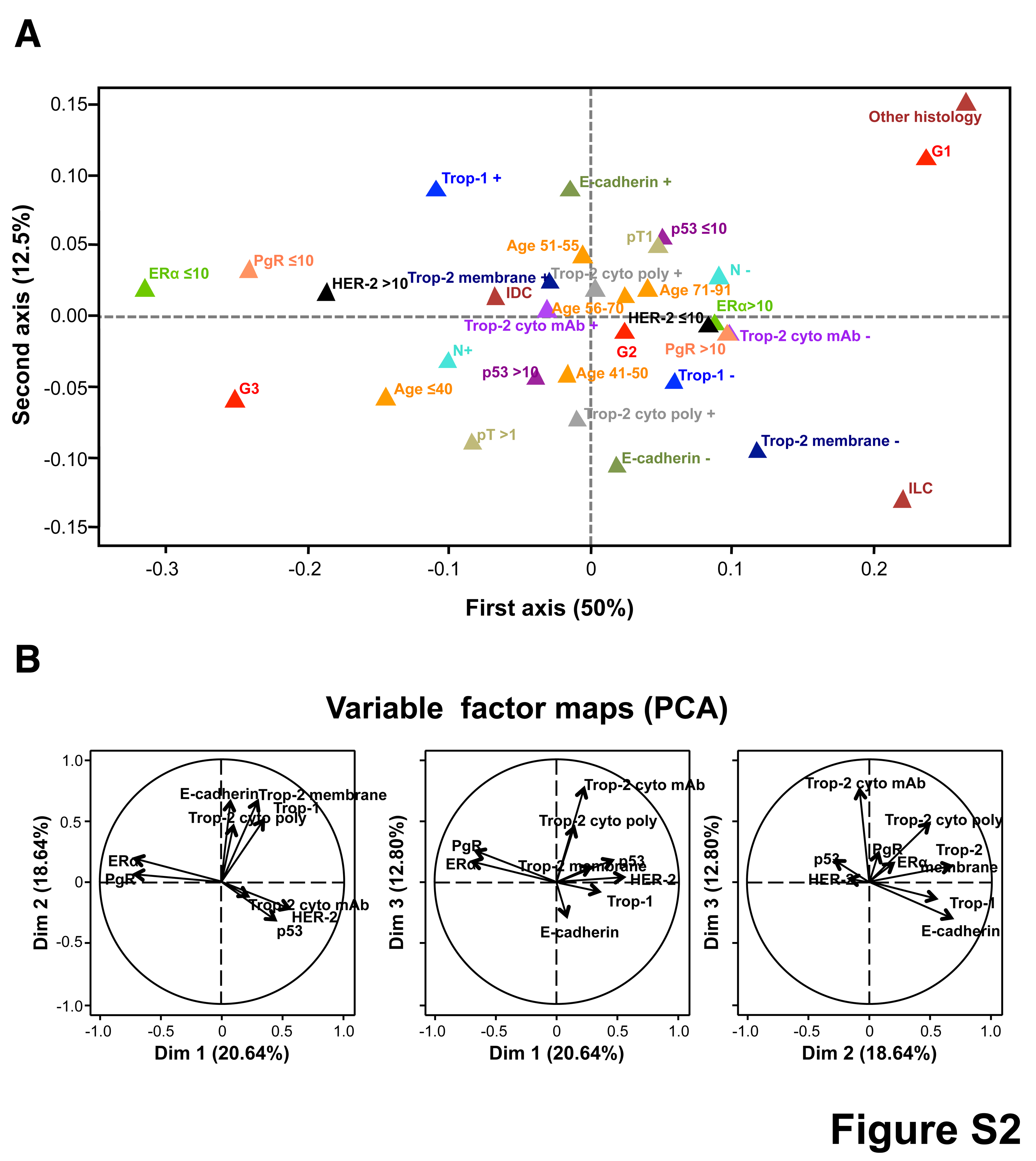

Supplement: Figure S2 — Association analysis for membrane and intracellular Trop-2. (TIF) [file pone.0096993.s002.tif]

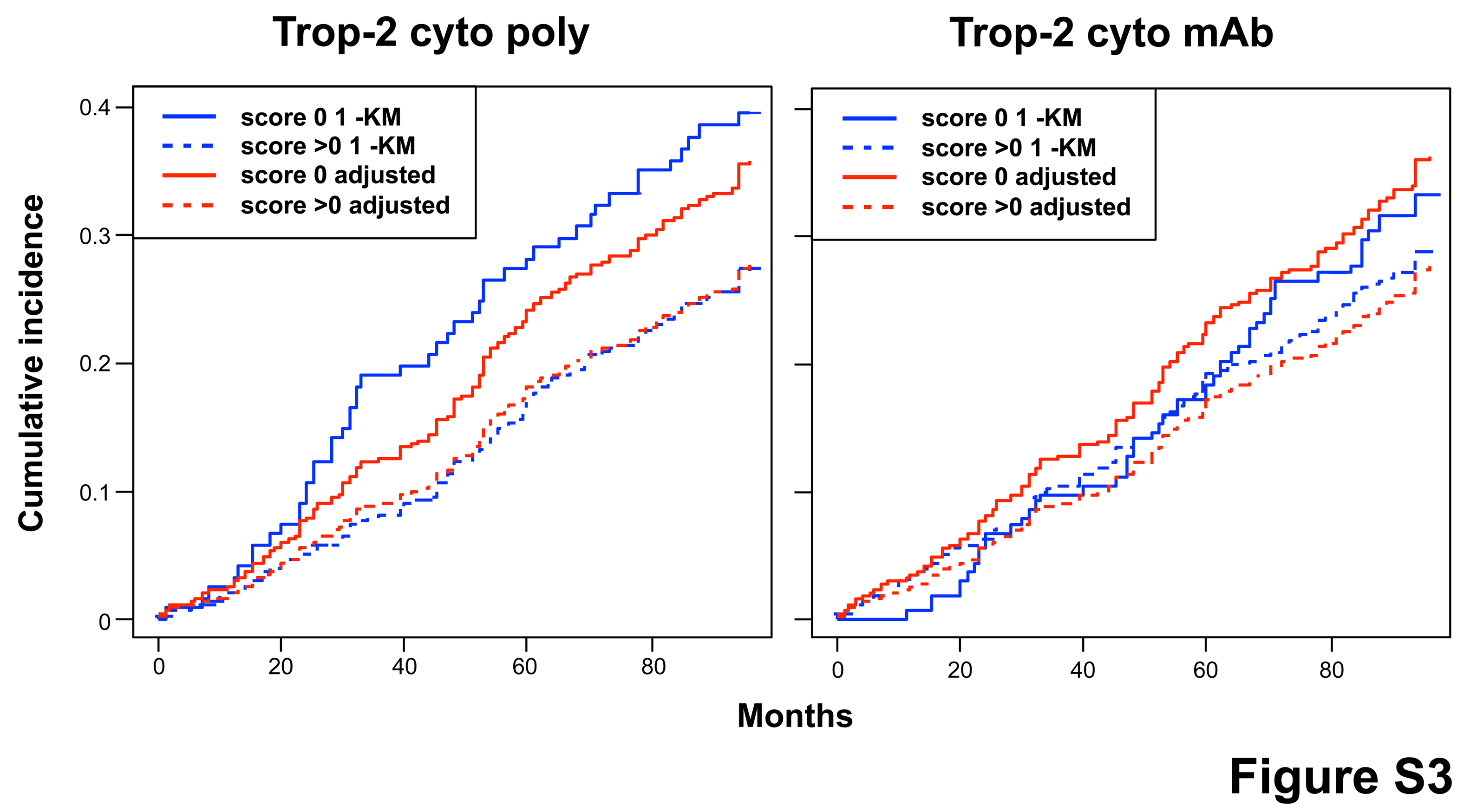

Supplement: Figure S3 — Adjusted impact on outcome for membrane and intracellular Trop-2. (TIF) [file pone.0096993.s003.tif]
