# Supplementary material for: Trop-2 Is a Determinant of Breast Cancer Survival
Source: PLoS One. 2014 May 13;9(5):e96993. doi: 10.1371/journal.pone.0096993 (PMC4019539; doi:10.1371/journal.pone.0096993)
Supplement: File S1 — This file contains Supporting Materials and Methods, Supporting Results, Supporting References, and Tables S1-S8. Table S1, Association between Trop-2 surface expression and tumor progression markers. Table S2, Frequency of tumor histotypes. Table S3, Intensity scores distribution. Table S4, Absolute frequency of first adverse events by lymph node status. Table S5, Absolute frequency of first adverse events by percentage of cells stained for intracellular Trop-2 – mAb detection. Table S6, Absolute frequency of first adverse events within 96 months after surgery by percentage of cells stained for intracellular Trop-2 – polyclonal antibody detection. Table S7, Absolute frequency of first adverse events by percentage of cells stained for membrane Trop-2. Table S8, Adherence to REMARK criteria (adapted from [23] in Supporting References). (DOC) [file pone.0096993.s005.doc]

**Supporting Information**

**Trop-2 is a determinant of breast cancer survival**

F. Ambrogi, M. Fornili, P. Boracchi, M. Trerotola, V. Relli, P. Simeone, R. La Sorda, R. Lattanzio, P. Querzoli, M. Pedriali, M. Piantelli, E. Biganzoli and S. Alberti

**Supporting Materials and Methods**

*Cell lines*

The breast cancer cell lines HBL100, MDA-MB415, MDA-MB468, MDA-MB453, MDA-MB361, MCF-7, T47D, MDA-MB231, SKBR3, ZR7530, ZR751, ovarian OVCA-432, colon HT-29 and KM12SM , prostate DU145 from ATCC ([www.atcc.org](http://www.atcc.org/)) were grown in RPMI 1640 medium supplemented with 10% FCS (Gibco, Life Technologies, Monza, Italy). Murine MTE 4-14 cells were cultured in DMEM supplemented with 10% FCS (Gibco).

*Antibodies*

Antibodies directed against Trop-2 have been described in the main text. Other antibodies were against Trop-1 (H99 mAb ), ERα (MU368-UC mAb, Biogenex), PgR (1A6, Ventana), HER-2/neu (Hercep-Test™, Dako, Glostrup, Denmark) , CD44, CD44v, COX-1, COX-2, E-cadherin (ECH-6, Medite, Castelnuovo Del Garda, Italy). Secondary Alexa Fluor-conjugated antibodies (633-goat anti-rabbit IgG, 546-donkey anti-goat IgG) were from Invitrogen. Negative controls were normal serum or isotype- matched antibodies.

*Flow cytometry*

Cell staining for flow cytometry was performed as described . Fluorescence analysis was performed on fluorescence-activated flow cytometers (Canto, FACStar, FACS Vantage, Becton Dickinson, Sunnyvale, CA). To improve the detection of transfectants stained with FITC-mAb, subtraction of cell autofluorescence and displacement of FITC-stained cells in the red channel were performed as described .

*Confocal microscopy*

Cells plated on glass coverslips were fixed with 4% paraformaldehyde in PBS for 20 min. Permeabilization and blocking were performed in medium with 10% FCS and 0.1% saponin . Live cells on glass coverslips were stained in medium with 10% FCS at 37 °C for 5 min, then fixed with 4% paraformaldehyde in PBS for 20 min. Slides were analyzed with an LSM-510 META (Zeiss) confocal microscope.

*Antibody-mediated capping*

Cells were detached from culture plates using trypsin, and incubated with primary antibodies against Trop-2 for 20 min on ice. After washing, cells were incubated with the secondary Alexa Fluor 488-conjugated antibodies for 10 min at 37 °C, to cross-link target molecules and to induce capping of the antigen-antibody complex . The ‘capped’ cells were then fixed with 1% paraformaldehyde for 10 min at room temperature.

*Confocal time-lapse microscopy*

Live cells cultured on glass slides were analyzed in Leibovitz’s F15 culture medium without phenol red and bicarbonate, supplemented with 10% FCS, 100 IU/ml penicillin, 100 µg/ml streptomycin (Euroclone), 25 mM HEPES and 2 mM N-acetyl-cysteine (Sigma), to reduce free-radical damage. Cells were observed using a LSM-510 META (Zeiss) confocal microscope. Images were captured at 30 s / 1 min intervals.

*Electron microscopy*

Cells were fixed in 4% formaldehyde, 0.05% glutaraldehyde, 0.15 M HEPES, pH 7.3, for 10 min at 37 °C, and post-fixed for 50 min in 4% paraformaldehyde, 0.15 M HEPES, pH 7.3, at room temperature, as previously described . Cationized gold, protein A–gold, and anti-rabbit gold antibodies (10 nm colloidal gold particles) were from British BioCell (Cardiff, UK).

*RT-PCR*

PCR reactions were performed in a Mastercycler EP (Eppendorf, Hamburg, Germany), with HotMaster Taq DNA Polymerase (Eppendorf) (95 °C, 2 min; 95 °C, 30 sec, 60 °C, 30 sec; 72 °C, 30 sec, 35 cycles). Quantitative real-time PCR reactions were performed in a LightCycler® apparatus using the LightCycler-DNA Master SYBR Green I kit (Roche Diagnostics). Amplification of GAPDH was used as an internal standard.

*Patients characteristics*

Patients were considered eligible according to the following criteria: pathologic stage T1 to T3, availability of at least 10 resected axillary lymph nodes, absence of synchronous bilateral tumors or any other malignancy before breast cancer diagnosis and up to 6 months after surgery, absence of distant metastases at diagnosis and up to 6 months after surgery, and no neo‑adjuvant therapy . At diagnosis, 393 patients were classified as node-negative (pN-) and 309 as node-positive (pN+). According to the treatment protocols applied, 335 of them received an adjuvant therapy. Patients’ clinical baseline and follow-up data (date and site of relapse, last follow-up time or date of death and cause of death) were obtained from the Ferrara Cancer Registry. Informed written consent was obtained from all patients and the protocol of this study was approved by the University of Ferrara Research Ethics Committee and by the board of the Ministry of the University and Research (“*Identification and validation of new markers of metastasizing phenotype of breast cancer*“, prot. MM06095812_006, year 2000)**.**

Data on patient age, histologic type, pathologic stage and grading were collected. Immunohistochemical assessment of routine biological markers including ERα and progesterone receptor (PgR), HER-2/neu and p53 was performed together with the evaluation of Trop-1, E-cadherin, and Trop-2, as indicated. Frequencies of tumor histotypes are detailed in Table S2. Intensity scores distributions are reported in Table S3.

*Tissue micro-array (TMA) construction*

TMA blocks were assembled as follows. A Tru-Cut needle (4 mm in internal diameter) was used to punch 3 mm-spaced holes in the recipient block. Whole-tumor sections stained with hematoxylin and eosin (H&E) were utilized to guide tumor punching in donor tumor blocks. Representative tumor areas were identified, tumor tissue cores were removed from donor blocks and transferred to the recipient block (24 samples per slide). The TMA was then incubated for 15 min at 37 °C to allow the tumor cores to adhere to the recipient block.

*Immunohistochemistry*

Consecutive 5 µm-thick sections were cut from the TMA and mounted on silanised slides. Slides were deparaffinised and rehydrated through graded alcohols to water. Endogenous peroxidases were blocked by incubation with 3% H_2_O_2_ for five minutes. IHC was performed with an automated immunostainer (Ventana NEXES, Medical System, Tucson, AZ). Slides were treated with 0.3% BSA in Tris-buffered saline, at room temperature, for 30 min, to reduce non-specific background staining. They were subsequently incubated at room temperature for 30 min with the appropriate primary antibodies. Slides treated with normal serum or isotype- matched antibodies were used as negative controls. The slides were then rinsed three times in Tris-buffered saline/0.05% Tween 20. Mouse monoclonal antibodies were detected by applying anti-mouse horseradish peroxidase-labeled polymer secondary antibodies (EnVision TM System, DAKO). E-cadherin was revealed using the Vectastain ABC peroxidase kit (Vector Laboratories, DBA Italia, Segrate, Italy) as secondary reagent . Staining for the Trop-2 mature form was performed after antigen retrieval by microwave at 750 W for 10 min in 1 M urea buffer (pH 8.0); sections were then incubated at RT with anti-Trop-2 polyclonal antibody (R&D) (40 min, 1:25 dilution). The LSAB kit (K0679, Dako) was used for signal amplification. After staining, slides were incubated for 10 min in 3,3’-diaminobenzidine (DAKO). Slides were then counterstained with Mayer’s hematoxylin and mounted with Immunomount (Shandon, Pittsburgh, PA).

All sections were independently examined by at least two pathologists (PQ, MP, RL, MP). For each tumor at least 400 cells were counted from different optical fields. Cases of disagreement were solved by joint re-evaluation.

*Statistical analysis*

The association between Trop-2 (membrane-associated or intracellular; continuous variables), and that of other clinico-biological markers, i.e. patient age, histologic type, grading, pT, lymph node status, ERα, PgR, HER-2/neu, p53, Trop-1 and E-cadherin was investigated through multiple correspondence analysis (MCA). MCA was used to visualize the association of both categorical and continuous variables using two dimensional plots , as MCA implies neither linearity nor specific distribution characteristics, and is useful to detect association of putative prognostic markers with clinical and pathological characteristics of the tumors.

The association between Trop-2 (membrane-associated or intracellular; continuous variables) and that of ERα, PgR, HER-2/neu, p53, Trop-1 and E-cadherin, was further investigated through principal component analysis (PCA) via three-dimensional biplots. Loadings of the first three principal components were plotted. Each variable was represented by an arrow starting from the origin. Angles between arrows were proportional to their correlation. Negatively correlated variables were characterized by opposite signs (+ and -) on the same side of the plot; positively correlated markers were identified by the same sign (+ and + or - and -). The variance explained by the first three principal components was used to evaluate the amount of information retained in the analysis.

**Supporting results**

*Association of Trop-2 with cancer progression determinants in vitro*

### Given the role of Trop-1/Ep-CAM and Trop-2 on tumor growth , we assessed whether Trops associated with major determinants of breast cancer progression, i.e. Her2, ERα, PgR, EGFR, CD44, CD44v, COX-l, COX-2, and with aggressive cancer subtypes (Table S1) . Trop-2 expression levels were found to be broadly heterogeneous. Notably, association was revealed with membrane Trop-1, CD44v and with ERα/PgR-negative cases. Prevalent association with distinct breast cancer subgroups (luminal, triple negative) was also identified. These findings were confirmed at the mRNA level (Table S1), consistent with dependence on distinct transcription regulatory networks .

*Patient case series*

Pre-menopausal cases represented 27.6% of the patients. Age distribution reflected common distribution profiles in aging Western countries ([www.worldlifeexpectancy.com/](http://www.worldlifeexpectancy.com/)). Major tumor histotypes were ductal (75.3%) and lobular (15.5%), with 18.1% grade 1 and 20.0% grade 3 cases ([www.wcrf.org/cancer_statistics/cancer_frequency.php](http://www.wcrf.org/cancer_statistics/cancer_frequency.php)). Lymph node diffusion was present in 44.2% of patients. The expression profiles of ERα, PgR, HER-2/neu, p53 and E-cadherin matched well those observed in other independent breast cancer case series .

*Categorical analysis*

Odds ratios, as obtained by multiple logistic regression, showed association of pAb-detected intracellular Trop-2 with hystotype and lymph node status, whereas the mAb-detected intracellular Trop-2 associated with pathological stage and E-cadherin, supporting the indication that these parameters correlate with diverse structure-function states of the Trop-2 molecules. Intracellular Trop-2 expression showed association with membrane Trop-1 expression. No association was found between intracellular Trop-2 expression and ERα, PgR, HER-2/neu and p53 status, supporting the indication that ‘intracellular Trop-2’ is an independent marker in breast cancer.

*Adjusted curves for death cumulative incidence*

Adjusted curves for death cumulative incidence for nil and positive scores of Trop-2 intra-cellular determination, as derived from the Cox model, were determined. The separation of the adjusted cumulative incidence curves was enhanced for the mAb-detected cytoplasmic Trop-2 (Figure S3, right). The separation of the adjusted cumulative incidence curves was statistically significant for the polyclonal antibody-detected cytoplasmic Trop-2 (Figure S2, left). No evidence for time-dependent effects for Trop-2 was found according to Schoenfeld residuals both in unadjusted and adjusted analysis.

**Supporting Movies.**

**Movie S1. Trop-2 capping by antibodies cross-linking.**

MTE 4-14 cells transfected with Trop-2-mRFP1 (red), treated with T16-Alexa 488 mAb (green). The time lapse covers a period of about 29 min. Binding of the mAb T16 is utilized to quantify binding kinetics to the transfected chimeric molecule and the Trop-2 capping. A cell membrane capping site is indicated by the arrowhead.

**Table S1 – Association between Trop-2 surface expression and tumor progression markers.**

| **Linea** | **Her2** | | **ERα** | **PgR** | **EGFR** | | **CD44** | **CD44v** | **Trop-1** | **Trop-2** | **COX-1** | **COX-2** | **tumor**  **phenotype** |
| --- | --- | --- | --- | --- | --- | --- | --- | --- | --- | --- | --- | --- | --- |
| **HBL100** | 1.1* | 0.8° |  |  | 27.0* | 46.8° | 47.1* | 1.1* | 0.3* | 9.0* | ++ | +/- |  |
| **MDA-MB415** | 7.1 | 2.1 | positive | negative | 3.6 | 0.2 | 33.5 | 34.0 | 82.0 | 126.0 | ND | ND | luminal 6% |
| **MDA-MB468** | 0.1 | 0.6 | negative | negative | 1025 | 2415 | 189.5 | 56.2 | 34.6 | 86.5 | - | + | basal A 82% |
| **MDA-MB453 !** | 14.4 | 14.2 | negative | negative | 1.6 | 0.6 | 0.3 | 0.0 | 90.0 | 68.8 | +++ | ++ | luminal 73% |
| **MDA-MB361** | 31.6 | 40.8 | positive | positive | 6.8 | 46.8 | 8.1 | 8.0 | 122 | 27.1 | +++ | +/- | luminal 4% |
| **MCF-7** | 1.9 | 1 | positive | positive | 10.9 | 4.7 | 6.2 | 9.5 | 142.0 | 74.4 | ++ | +/-- | luminal 57% |
| **T47D** | 4.8 | 0.5 | positive | positive | 6.4 | 1.4 | 10.7 | 15.3 | 61.7 | 55.4 | + | +/- | luminal 53% |
| **MDA-MB231** | 0.9 | 0.8 | negative | negative | 65.0 | 91.8 | 124.8 | 1.2 | 1.2 | 14.4 | ++ | ++/+ | basal B 50% |
| **SKBR3** | 88 | 46.9 | negative | negative | 28.3 | 50.0 | 9.8 | 4.2 | 96.1 | 115.8 | ++++ | +/- | luminal 35% |
| **ZR7530** | 129 | 62.7 | positive | negative | 0.6 | 0.7 | 58.4 | 44.8 | 98.1 | 235.0 | ND | ND |  |
| **ZR751** | 16 | 2.4 | positive | positive | 10.9 | 4.4 | 32.0 | 26.6 | 205.7 | 97.3 | + | +/- | luminal 6% |
|  |  |  |  |  |  |  |  |  |  |  |  |  |  |
| **DU145** # | 2.1 |  |  |  | 66.4 |  | 115.1 | 2.6 | 38.6 | 49.0 | ND | ND |  |

*: protein expression levels were determined by flow cytometry, as expressed in mean fluorescence units; °: mRNA expression levels were determined by real time RT-PCR, as normalized versus Her2 expression in MCF-7 cells; light gray: breast cancer phenotype; dark gray: high levels of Trop-2; !: amplified *HER2* gene; #: prostate. The DU145 prostate cancer cell line was used as a Trop-2-dependent tumor progression model .

**Table S2 – Frequency of tumor histotypes.**

| **Histologic Type** | **All**  **Freq. (*%*)** | **N+**  **Freq. (*%*)** | **N-**  **Freq. (*%*)** |
| --- | --- | --- | --- |
|  |  |  |  |
| Cribriform | 6 (0.9) | 0 (0.0) | 6 (1.5) |
| Ductal | 527 (75.1) | 254 (82.2) | 273 (69.5) |
| Special histotypes | 3 (0.4) | 1 (0.3) | 2 (0.5) |
| Lobular | 109 (15.5) | 44 (14.2) | 65 (16.6) |
| Mucinous | 18 (2.6) | 3 (1.0) | 15 (3.8) |
| Papillar | 8 (1.1) | 2 (0.7) | 6 (1.5) |
| Tubular | 24 (3.4) | 3 (1.0) | 21 (5.3) |
| Medullary | 7 (1.0) | 2 (0.6) | 5 (1.3) |
|  | 702 (100.0) | 309 (100.0) | 393 (100.0) |

**Table S**3 – Intensity scores distribution.

| **Variable** | **All**  **Freq. (*%*)** | **N+**  **Freq. (*%*)** | **N-**  **Freq. (*%*)** |
| --- | --- | --- | --- |
| ERα | | | |
| 0% | 34 (*5.8*) | 16 (*6.0*) | 18 (*5.5*) |
| 1-10% | 93 (*15.8*) | 54 (*20.4*) | 39 (*12.0*) |
| >10% | 463 (*78.6*) | 195 (*73.6*) | 268 (*82.5*) |
|  | 590 (*100.0*) | 265 (*100.0*) | 325 (*100.0*) |
| PgR | | | |
| 0% | 40 (*6.8*) | 18 (*6.8*) | 22 (*6.8*) |
| 1-10% | 133 (*22.7*) | 68 (*25.9*) | 65 (*20.2*) |
| >10% | 412 (*70.5*) | 177 (*67.3*) | 235 (*73.0*) |
|  | 585 (*100.0*) | 263 (*100.0*) | 322 (*100.0*) |
| Trop-2 cyto (mAb) | | | |
| 0 | 165 (*26.6*) | 72 (*26.3*) | 93 (*26.9*) |
| 1+ | 203 (*32.7*) | 86 (*31.3*) | 117 (*33.8*) |
| 2+ | 130 (*21.0*) | 58 (*21.2*) | 72 (*20.8*) |
| 3+ | 122 (*19.7*) | 58 (*21.2*) | 64 (*18.5*) |
|  | 620 (*100.0*) | 274 (*100.0*) | 346 (*100.0*) |
| Trop-2 cyto (pAb) | | | |
| 0 | 133 (*21.6*) | 52 (*18.6*) | 81 (*24.0*) |
| 1+ | 155 (*25.1*) | 69 (*24.7*) | 86 (*25.4*) |
| 2+ | 290 (*47.0*) | 140 (*50.2*) | 150 (*44.4*) |
| 3+ | 39 (*6.3*) | 18 (*6.5*) | 21 (*6.2*) |
|  | 617 (*100.0*) | 279 (*100.0*) | 338 (*100.0*) |
| Trop-2 membrane (pAb) | | | |
| 0 | 149 (*22.4*) | 54 (*18.3*) | 95 (*25.7*) |
| 1+ | 236 (*35.5*) | 105 (*35.6*) | 131 (*35.4*) |
| 2+ | 187 (*28.1*) | 93 (*31.5*) | 94 (*25.4*) |
| 3+ | 93 (*14.0*) | 43 (*14.6*) | 50 (*13.5*) |
|  | 665 (*100.0*) | 295 (*100.0*) | 370 (*55.6*) |
| Trop-1 | | | |
| 0 | 428 (*66.7*) | 187 (*65.8*) | 241 (*67.4*) |
| 1+ | 99 (*15.4*) | 41 (*14.4*) | 58 (*16.2*) |
| 2+ | 60 (*9.3*) | 28 (*9.9*) | 32 (*8.9*) |
| 3+ | 55 (*8.6*) | 28 (*9.9*) | 27 (*7.5*) |
|  | 642 (*100.0*) | 284 (*100.0*) | 358 (*100.0*) |
| E-cadherin | | | |
| 0 | 304 (*48.4*) | 131 (*46.7*) | 173 (*49.7*) |
| 1+ | 164 (*26.1*) | 71 (*25.4*) | 93 (*26.7*) |
| 2+ | 120 (*19.1*) | 57 (*20.4*) | 63 (*18.1*) |
| 3+ | 40 *(6.4*) | 21 *(7.5*) | 19 (*5.5*) |
|  | 628 (*100.0*) | 280 (*100.0*) | 348 (*100.0*) |

IHC-detected parameters were categorized as described in Material and patients. Absolute numbers are indicated. Percentages are in brackets.

**Table S4 – Absolute frequency of first adverse events by lymph node status.**

|  | **Distant**  **metastases** | **Local**  **relapse** | **Contra-lateral tumor** | **Other**  **malignancy** | **Death** | **Total ^a^** |
| --- | --- | --- | --- | --- | --- | --- |
| **N+** (*n=309*) | 74 | 26 | 6 | 13 | 47 | 166 |
| **N-** (*n=393*) | 36 | 26 | 8 | 20 | 49 | 139 |
| **Total**  (*n=702*) | 110 | 52 | 14 | 33 | 96 | 305 |

^a^: within 96 months after surgery. At least a fraction of deaths without evidence of recurrence may be related to the high prevalence of elderly patients in the case series analysed.

**Table S5 – Absolute frequency of first adverse events by percentage of cells stained for intracellular Trop-2 – mAb detection.**

|  | **Distant**  **metastases** | **Local**  **relapse** | **Contra-lateral tumor** | **Other**  **malignancy** | **Death** | **Total ^a^** |
| --- | --- | --- | --- | --- | --- | --- |
| **0-5** (*n=166*) | 27 | 16 | 1 | 11 | 28 | 83 |
| **6-85**  (*n=246*) | 37 | 16 | 5 | 13 | 38 | 109 |
| **86-100**  (*n=208*) | 31 | 12 | 6 | 7 | 19 | 75 |
| **Total**  (*n=620*) | 95 | 44 | 12 | 31 | 85 | 267 |

^a^: within 96 months after surgery.

**Table S6 – Absolute frequency of first adverse events within 96 months after surgery by percentage of cells stained for intracellular Trop-2 – polyclonal antibody detection.**

|  | **Distant**  **metastases** | **Local**  **relapse** | **Contra-lateral tumor** | **Other**  **malignancy** | **Death** | **Total ^a^** |
| --- | --- | --- | --- | --- | --- | --- |
| **0-5** (*n=140*) | 23 | 11 | 3 | 6 | 22 | 65 |
| **6-85**  (*n=293*) | 43 | 25 | 7 | 13 | 41 | 129 |
| **86-100**  (*n=184*) | 28 | 10 | 2 | 9 | 20 | 69 |
| **Total**  (*n=617*) | 94 | 46 | 12 | 28 | 83 | 263 |

^a^: within 96 months after surgery.

**Table S7 – Absolute frequency of first adverse events by percentage of cells stained for membrane Trop-2.**

|  | **Distant**  **metastases** | **Local**  **relapse** | **Contra-lateral tumor** | **Other**  **malignancy** | **Death** | **Total ^a^** |
| --- | --- | --- | --- | --- | --- | --- |
| **0-5** (*n=212*) | 32 | 19 | 8 | 11 | 22 | 92 |
| **6-85**  (*n=395*) | 61 | 26 | 5 | 18 | 62 | 172 |
| **86-100**  (*n=58*) | 8 | 3 | 0 | 2 | 11 | 24 |
| **Total**  (*n=665*) | 101 | 48 | 13 | 31 | 95 | 288 |

^a^: within 96 months after surgery.

**Table S8 – Adherence to REMARK criteria (adapted from** )

| **Item to be reported** | | **Page no.** |
| --- | --- | --- |
| **INTRODUCTION** | |  |
| 1 | State the marker examined, the study objectives, and any pre-specified hypotheses. | “Introduction” section. |
| **MATERIALS AND METHODS** | |  |
| *Patients* | |  |
| 2 | Describe the characteristics (e.g., disease stage or co-morbidities) of the study patients, including their source and inclusion and exclusion criteria. | Table 1 and Table S2; “Patients characteristics” paragraph in the “Supporting Materials and Methods” section; “Patient case series” in the “Supporting Results” section. |
| 3 | Describe treatments received and how chosen (e.g., randomized or rule-based). | “Patients characteristics” paragraph in the “Supporting Materials and Methods” section. |
| *Specimen characteristics* | |  |
| 4 | Describe type of biological material used (including control samples) and methods of preservation and storage. | “TMA construction”  and “Immunohistochemistry” paragraphs in “Supporting Materials and Methods” section; Table S2. |
| *Assay methods* | |  |
| 5 | Specify the assay method used and provide (or reference) a detailed protocol, including specific reagents or kits used, quality control procedures, reproducibility assessments, quantitation methods, and scoring and reporting protocols. Specify whether and how assays were performed blinded to the study endpoint. | “Immunohistochemistry” paragraphs in “Materials and Methods” and “Supporting Materials and Methods” sections. |
| *Study design* | |  |
| 6 | State the method of case selection, including whether prospective or retrospective and whether stratification or matching (e.g., by stage of disease or age) was used. Specify the time period from which cases were taken, the end of the follow-up period, and the median follow-up time. | “Patients” paragraph in the “Materials and Methods” section; Table 1 and Table S2. |
| 7 | Precisely define all clinical endpoints examined. | “Statistical analysis” paragraph in the “Materials and Methods” section. |
| 8 | List all candidate variables initially examined or considered for inclusion in models. | “Statistical analysis” paragraph in the “Materials and Methods” and “Supporting Materials and Methods” sections. |
| *Statistical analysis methods* | |  |
| 9 | Specify all statistical methods, including details of any variable selection procedures and other model-building issues, how model assumptions were verified, and how missing data were handled. | “Statistical analysis” paragraph in the “Materials and Methods” and “Supporting Materials and Methods” sections. |
| 10 | Clarify how marker values were handled in the analyses; if relevant, describe methods used for cutpoint determination. | “Statistical analysis” paragraph in the “Materials and Methods” and “Supporting Materials and Methods” sections. |
| **RESULTS** | |  |
| *Data* | |  |
| 11 | Describe the flow of patients through the study, including the number of patients included in each stage of the analysis (a diagram may be helpful) and reasons for dropout. Specifically, both overall and for each subgroup extensively examined report the numbers of patients and the number of events. | Table 1. |
| 12 | Report distributions of basic demographic characteristics (at least age and sex), standard (disease-specific) prognostic variables, and tumor marker, including numbers of missing values. | Tables 1, S2, S3. |
| *Analysis and presentation* | |  |
| 13 | Show the relation of the marker to standard prognostic variables. | Tables 1-2, S4-S7. Figures 4-5, S2-S3. |
| 14 | Present univariable analyses showing the relation between the marker and outcome, with the estimated effect (e.g., hazard ratio and survival probability). Preferably provide similar analyses for all other variables being analyzed. For the effect of a tumor marker on a time-to-event outcome, a Kaplan-Meier plot is recommended. | Figures 4-5, S3. Table 2. |
| 15 | For key multivariable analyses, report estimated effects (e.g., hazard ratio) with confidence intervals for the marker and, at least for the final model, all other variables in the model. | Figure S2. |
| 16 | Among reported results, provide estimated effects with confidence intervals from an analysis in which the marker and standard prognostic variables are included, regardless of their statistical significance. | “Association of membrane Trop-2 with tumor progression determinants in patients” paragraph in the “Results” section. |
| **DISCUSSION** | |  |
| 17 | Interpret the results in the context of the pre-specified hypotheses and other relevant studies; include a discussion of limitations of the study. | “Discussion” section. |
| 18 | Discuss implications for future research and clinical value. | “Discussion” section. |

**Supporting References**

1. Morikawa K, Walker SM, Jessup JM, Fidler IJ (1988) In vivo selection of highly metastatic cells from surgical specimens of different primary human colon carcinomas implanted into nude mice. Cancer Res 48: 1943-1948.

2. Naquet P, Lepesant H, Luxembourg A, Brekelmans P, Devaux C, et al. (1989) Establishment and characterization of mouse thymic epithelial cell lines. Thymus 13: 217-226.

3. Mattes MJ, Cairncross JG, Old LJ, Lloyd KO (1983) Monoclonal antibodies to three widely distributed human cell surface antigens. Hybridoma 2: 253-264.

4. Tinari N, Lattanzio R, Natoli C, Cianchetti E, Angelucci D, et al. (2006) Changes of topoisomerase IIalpha expression in breast tumors after neoadjuvant chemotherapy predicts relapse-free survival. Clin Cancer Res 12: 1501-1506.

5. Dell'Arciprete R, Stella M, Fornaro M, Ciccocioppo R, Capri MG, et al. (1996) High-efficiency expression gene cloning by flow cytometry. J Histochem Cytochem 44: 629-640.

6. Alberti S, Bucci C, Fornaro M, Robotti A, Stella M (1991) Immunofluorescence analysis in flow cytometry: better selection of antibody-labeled cells after fluorescence overcompensation in the red channel. J Histochem Cytochem 39: 701-706.

7. Alberti S, Parks DR, Herzenberg LA (1987) A single laser method for subtraction of cell autofluorescence in flow cytometry. Cytometry 8: 114-119.

8. Polishchuk RS, Polishchuk EV, Marra P, Alberti S, Buccione R, et al. (2000) Correlative light-electron microscopy reveals the saccular-tabular ultrastructure of carriers operating between the Golgi apparatus and the plasma membrane. J Cell Biol 148: 45-58.

9. Levy S, Shoham T (2005) The tetraspanin web modulates immune-signalling complexes. Nat Rev Immunol 5: 136-148.

10. Ambrogi F, Biganzoli E, Querzoli P, Ferretti S, Boracchi P, et al. (2006) Molecular subtyping of breast cancer from traditional tumor marker profiles using parallel clustering methods. Clin Cancer Res 12: 781-790.

11. Elston CW, Ellis IO (1991) Pathological prognostic factors in breast cancer. I. The value of histological grade in breast cancer: experience from a large study with long-term follow-up. Histopathology 19: 403-410.

12. Querzoli P, Coradini D, Pedriali M, Boracchi P, Ambrogi F, et al. (2010) An immunohistochemically positive E-cadherin status is not always predictive for a good prognosis in human breast cancer. Br J Cancer 103: 1835-1839.

13. Querzoli P, Pedriali M, Rinaldi R, Lombardi AR, Biganzoli E, et al. (2006) Axillary lymph node nanometastases are prognostic factors for disease-free survival and metastatic relapse in breast cancer patients. Clin Cancer Res 12: 6696-6701.

14. Greenacre MJ (1994) Theory and Applications of Correspondence Analysis. New York: Academic Press.

15. Biganzoli E, Boracchi P, Daidone MG, Gion M, Marubini E (1998) Flexible modelling in survival analysis. Structuring biological complexity from the information provided by tumor markers. Int J Biol Markers 13: 107-123.

16. Lin CW, Liao MY, Lin WW, Wang YP, Lu TY, et al. (2012) Epithelial cell adhesion molecule regulates tumor initiation and tumorigenesis via activating reprogramming factors and epithelial-mesenchymal transition gene expression in colon cancer. J Biol Chem 287: 39449-39459.

17. Trerotola M, Cantanelli P, Guerra E, Tripaldi R, Aloisi AL, et al. (2013) Up-regulation of Trop-2 quantitatively stimulates human cancer growth. Oncogene 32 222-233.

18. Hanahan D, Weinberg RA (2011) Hallmarks of cancer: the next generation. Cell 144: 646-674.

19. Guerra E, Trerotola M, Aloisi AL, Tripaldi R, Vacca G, et al. (2013) The Trop-2 signalling network in cancer growth. Oncogene 32: 1594-1600.

20. De Vita VT, Lawrence TS, Rosenberg SA (2008) De Vita, Hellman & Rosenberg's Cancer: Principles & Practice of Oncology, 8th Edition; De Vita VT, Hellman S, Rosenberg SA, editors. Philadelphia: Lippincott Williams & Wilkins.

21. Trerotola M, Li J, Alberti S, Languino LR (2012) Trop-2 inhibits prostate cancer cell adhesion to fibronectin through the β1 integrin-RACK1 axis. J Cell Physiol 227: 3670-3677.

22. Trerotola M, Jernigan D, Liu Q, Siddiqui J, Fatatis A, et al. (2013) Trop-2 promotes prostate cancer metastasis by modulating β1 integrin functions. Cancer Res 73: 3155-3167.

23. Altman DG, McShane LM, Sauerbrei W, Taube SE (2012) Reporting Recommendations for Tumor Marker Prognostic Studies (REMARK): explanation and elaboration. PLoS Med 9: e1001216.

**Supporting Figure Legend**

**Figure S1 - Trop-2 subcellular retention.**

Cancer cell membrane versus intracytoplasmic Trop-2 deposits were assessed. Breast MCF-7 cancer cells were stained with the mAb T16 as (**A**) fixed/permeabilized cells versus (**B**) living counterparts. Trop-1/Ep-CAM was used as benchmark for comparative profiling.

(**A**) Distinct localization at cell-cell and cell-substrate binding sites was revealed, together with intracellular granular deposits in most cancer cells. These were shown to contain Trop-2 (white arrowheads), but rarely Trop-1. Z-stacking allowed to formally distinguish *bona fide* Trop-2 intracellular deposits from cell surface-associated membrane organelles, e.g. basal podosomes, cell surface macrovilli.

(**B**) Islands-packed living cells were shown to be accessible to binding by 2EF anti-Trop-2 mAb, for transduction of cell signals, capping and internalization (Figures 1, 2 and Movie S1).

**Figure S2 – Association analysis for membrane and intracellular Trop-2.**

(**A**) Multiple correspondence analysis. The variables projected on the plot are categorized as in Table 1. ILC: Lobular carcinoma; IDC: Ductal carcinoma; the few tumors with cribriform, mucinous, papillar or tubular histology were grouped together as “other histology”; pT >1 groups pT2 and pT3; N+: positive nodal status; N-: negative nodal status; Trop-1-: membrane Trop-1 score 0; Trop-1+: membrane Trop-1 score 1-12; Trop-2-: membrane Trop-2 score 0; Trop-2+: membrane Trop-2 score 1-12; Trop-2 cytoplasmic polyclonal score 0: Trop-2 cyto poly -; Trop-2 cyto poly +: Trop-2 cytoplasmic polyclonal score 1-12; Trop-2 cyto mAb -: Trop-2 cytoplasmic monoclonal score 0; Trop-2 cyto mAb +: Trop-2 cytoplasmic monoclonal score 1-12; E-cadherin -: E-cadherin score 0; E-cadherin +: E-cadherin score 1-12; G1, G2, G3: grade 1, 2 or 3, respectively. Expression of p53, ERα, PgR and Her-2 was categorized with a cut-off of ≤ or >10% positive cells (Table 1). Age was as indicated.

(**B**) Biplot analysis for membrane and intracellular Trop-2. Variable factors maps were generated by PCA. Membrane Trop-1: Trop-1; Trop-2 cytoplasmic polyclonal: Trop-2 cyto poly; Trop-2 cytoplasmic monoclonal: Trop-2 cyto mAb. Expression of p53, ERα, PgR, Her-2, E-cadherin and membrane Trop-2 were as indicated.

**Figure S3 – Adjusted impact on outcome for membrane and intracellular Trop-2.**

Cumulative incidence estimates were obtained as 1-Kaplan-Meier curves for Trop-2 expression sub-groups (cell membrane, mAb-detected intracellular or polyclonal-detected intracellular). Trop-2 expression was categorized according to intensity scores (0 or >0). Adjusted cumulative incidence of relapse was estimated as described in Material and patients. Adjusted curves are in red, unadjusted curves are in blue.
